# Crosstalk between Circadian Rhythm Dysregulation and Tumorigenesis, Tumor Metabolism and Tumor Immune Response

**DOI:** 10.14336/AD.2024.0533

**Published:** 2025-06-07

**Authors:** Yifei Zhu, Yao Zheng, Rongyu Dai, Xuyu Gu

**Affiliations:** ^1^Department of Oncology, Shanghai Medical College of Fudan University, Shanghai, China.; ^2^Department of Pathology, Fudan University Shanghai Cancer Center, Shanghai, China.; ^3^Cancer Institute, Fudan University Shanghai Cancer Center, Shanghai, China.; ^4^Institute of Pathology, Fudan University, Shanghai, China.; ^5^Shanghai Institute of Biochemistry and Cell Biology, Chinese Academy of Sciences, University of the Chinese Academy of Sciences, Shanghai, China.; ^6^School of Medicine, Southeast University, Nanjing, China.; ^7^Department of Oncology, Shanghai Pulmonary Hospital, School of Medicine, Tongji University, Shanghai, China.

**Keywords:** circadian rhythm, tumorigenesis, tumor metabolism, immune cells, immune response, tumor therapy

## Abstract

Circadian rhythm is a self-regulating 24-hour system that synchronizes with the day and night cycle in organisms. The regulation of this system is controlled by clock genes, which function to harmoniously express molecular levels that facilitate the orderly coordination of various cellular processes, such as sleep, metabolism, endocrine function, cell proliferation and immunity. The root cause of tumorigenesis is that the body loses its normal regulation of cell growth at the genetic level. Long-term disruptions in circadian rhythms caused by factors such as shift work, jet lag, and unstable sleep patterns can impact cellular health, leading to various health problems, including cancer. Circadian rhythm controls most cellular functions related to cancer progression, which has a significant impact on the ability of immune cells to detect cancer cells and promote their clearance and has crucial implication for future tumor immunotherapy. This article aims to review the crosstalk between dysregulation of circadian rhythm and tumorigenesis, tumor metabolism, and immune response. Additionally, we discuss the role of circadian rhythm disruption in tumor therapy, highlighting its potential to optimize treatment timing and improve therapeutic outcomes.

## 1. Introduction

The human body operates on a finely tuned schedule governed by a unique biological clock, known as the circadian rhythm. This internal clock, regulated by specific genes, orchestrates a central clock in the brain that responds to environmental cues and synchronizes subordinate clocks in peripheral tissues through mechanisms tied to the day-night cycle. However, in our modern society, disruptions to this circadian rhythm, referred to as circadian rhythm dysregulation (CRD), have become increasingly common. This dysregulation poses significant threats to overall well-being, leading to adverse health outcomes, including metabolic, immune, and proliferative diseases [1, 2]. A robust body of epidemiological and laboratory evidence has established a link between the biological clock and cancer [3], but the precise molecular mechanism underlying this association has yet to be comprehensively explained.

Findings from several research endeavors suggest that disruptions to the biological circadian rhythm, resulting from night shift work and exposure to light, have a close relationship with hormone-dependent cancers [4-6]. Early studies have shown that the biological clock can influence regulatory processes crucial to cancer development by coupling transcription and metabolism, thereby regulating cell differentiation and proliferation [1, 3, 7].

Core clock and non-clock transcription factors (TF) regulate proliferative metabolism and systematic communication between tumor and host cells in a coordinated manner [8]. Emerging evidence suggests that the biological clock controls metabolism and is a special feature of cancer metabolism [9]. The disturbance of circadian rhythm leads to disordered chromatin remodeling [2, 10], which indicates that abnormal metabolism in cancer may also be the result of a biological clock disorder.

In experimental models and cancer patients, circadian rhythms substantially augment anticancer drug resistance [11, 12]. An enhancement in efficacy was observed when drugs were administered during the optimal time periods, when the treatment is most effective and well-tolerated by patients. Conversely, when anticancer drugs are administered during the peak of their toxicity, the host's circadian clock is disturbed [13], chiefly because of the inherent dysregulation of the circadian rhythm in tumor cells, while healthy tissue rhythms remain intact. The immune system adapts to circadian rhythms, and both dendritic cells and lymphocytes are required to respond to these rhythms for proper cellular migration [14]. The absence of circadian rhythm implies the absence of peak immune activity [14, 15]. Given this intricate relationship between the circadian rhythm and disease processes, the concept of chronotherapy has emerged as a promising strategy in optimizing treatment outcomes [16-18]. Chronotherapy involves the timing of medication administration to align with the body's biological rhythms, thereby enhancing drug efficacy and minimizing toxicity [19]. This approach has shown potential in cancer therapy, where the timing of drug delivery can significantly influence the therapeutic index [20]. By aligning treatment with the body's circadian rhythms, chronotherapy aims to maximize the effectiveness of drugs while reducing adverse effects, providing a novel avenue for improving patient outcomes. Our present focus delves into the interplay between CRD and tumorigenesis, tumor metabolism, and immune response, while underscoring the potential use of circadian rhythm in tumor therapy.

## 2. Circadian rhythm

The biological clock is a physiological rhythm system that regulates various behavioral activities and physiological functions within the living body via a self-regulating function and synchronization mechanism. The clock system that controls circadian rhythm in mammals is referred to as the circadian biological clock, which is primarily composed of central biological clock system and peripheral biological clock system [21]. The peripheral clock effectively oversees a multitude of molecular and cellular processes through various levels of regulation [22]. It is notable that a significant proportion of the genome is under the control of this clock, with over half of the protein-coding genes exhibiting varying diurnal oscillations within the tissue [23]. Furthermore, the regulation of the circadian rhythm of cellular activity extends beyond transcription and involves the rhythmic modulation of critical post-transcriptional processes such as RNA splicing, protein translation, and post-translation processing [24]. At the molecular level, circadian rhythms are the result of autonomous rhythms generated by oscillating clock genes [25]. The circadian rhythm system is a crucial regulatory system governing both bodily health and metabolism. It plays a vital role in regulating fundamental functions, including but not limited to gene expression, hormone release, energy consumption, cell growth, secretion, and metabolism [26, 27]. Over the years, numerous studies have indicated a significant correlation between metabolic homeostasis and the regular biological clock [28, 29]. Circadian rhythm participates in the metabolism of nutrients such as sugar and fat and in the metabolic regulation of various systems of the body [30-32]. Given that clock genes exhibit significant involvement in the regulation of neuroendocrine, immune, and cell cycle processes, with a notable impact on the cellular proliferation cycle and apoptosis. This close association with tumorigenesis and tumor metabolism further emphasizes its critical role in the maintenance of cellular homeostasis [33-36].

### 2.1 Molecular circuit of circadian rhythm

During the 1970s, Benzer and Konopka reported the isolation of one single-gene mutant in Drosophila that dramatically altered circadian rhythms in activity. In-depth research was conducted, the gene was designated as Period (*Per*) [37]. The protein encoded by *Per* functions as an inhibitor of its own transcription, leading to the emergence of rhythm [38]. Subsequently, the scientific community identified the Circadian motorcycle output kaput (Clock) gene, which is induced by *Per* and plays a significant role in mammalian organisms. This discovery highlights the role of activators in driving the expression of repressive genes, thereby creating a negative feedback loop that is conserved from Drosophila to humans [39]. The core clock mechanism consists of a self-regulatory network consisting of transcription-translation feedback loops (TTFL) incorporating both positive and negative feedback [39]. In terms of transcription, the circadian clock is facilitated by positive regulators within the loop. Basic helix-loop-helix heterodimer transcription factors (CLOCK/BMAL1 or BMAL1/NPAS2) are responsible for regulating the expression of pivotal circadian genes, including cryptochrome (*Cry1* and *Cry2*) and period (*Per1*, *Per2* and *Per3*) genes, which function as negative regulators of the circadian circuitry [40-42]. *Cry* and *Per* forms a transcriptional repressor complex in the nucleus to inhibit CLOCK/BMAL1 activity, thus creating a negative feedback loop to control the clock. BMAL1 (brain and muscle ARNT-like protein 1) is also rhythmically controlled through its own transcriptional targets. Feedback timing is regulated through post-transcriptional modifications (e.g., splicing and translation), particularly post-translational modifications.

**Figure 1.**
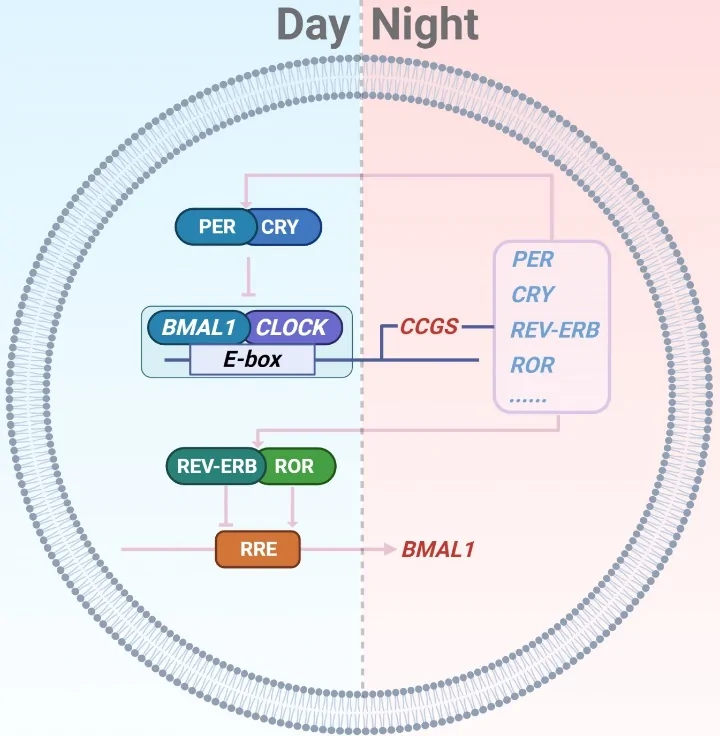
**Molecular composition of mammalian circadian clock**. The mammalian circadian clock is a time-delayed transcriptional-translational feedback loop operating over 24 hours. The core circadian machinery includes the basic helix-loop-helix (bHLH) transcription factors CLOCK and BMAL1, regulating rhythmic expression of approximately 10-15% of genes via E-Box sequences. During the daytime, CLOCK-BMAL1 drives the transcription of core clock and clock-controlled genes (CCGs), while at night, PER and CRY inhibit this transcription. Light exposure induces PER expression in the suprachiasmatic nucleus (SCN) and other brain regions immediately. In peripheral tissues, PER expression is delayed by several hours relative to the SCN, ensuring synchronization with the central clock. Additionally, nuclear receptors RORα and REV-ERBα provide an extra layer of regulation by activating and repressing BMAL1 transcription, respectively.

A prevalent regulatory pattern involves cyclic phosphorylation and degradation of biological clock constituents. These events are typically executed via the ubiquitin-proteasome system. The fundamental signaling pathway is fortified by the stimulation of REV-ERbα and ROR (Retinoid-related orphan receptor) α by CLOCK-BMAL1, which is integrated into additional transcriptional feedback mechanisms. Other transcription factors, such as USF1 and Dec1-Dec2, exert a feedback regulation over CLOCK activity [37, 41] (Fig. 1). RORs act as positive regulators by activating the transcription of BMAL1, while REVERBs function as negative regulators by repressing BMAL1 transcription [43]. This antagonistic interaction helps to establish and maintain the robustness of circadian rhythms. For example, RORα promotes BMAL1 expression, enhancing the transcription of clock-controlled genes [44], whereas REVERBα inhibits BMAL1 expression, thus dampening the circadian oscillations when needed [45]. The significance of the major clock regulators highlights the pivotal role of CLOCK/BMAL1 in modulating the expression of roughly 10% of clock-controlled genes that govern various molecular, biochemical, and physiological activities. Furthermore, protein stability is regulated by post-translational modifications of *Cry* and *Per*, whereas self-regulatory clock feedback loops can be influenced by repressors that control *Cry* or *Per*. These overlapping mechanisms confer daily rhythmicity in cellular, metabolic, and physiological functions to maintain homeostasis. Recent studies have shown that alterations in clock regulators are associated with cancer phenotypes [46-50].

## 3. CRD and tumorigenesis

The proliferation rhythm of tumor cells follows a different cycle pattern than that of normal tissue [51-54]. One of the characteristics of malignant tumors is the uncontrolled and disordered proliferation of cells, which is necessarily reflected in the disruption of the temporal dimension. Circadian clock genes are closely associated with tumorigenesis because they are closely related to neuroendocrine, immune, and cell cycle regulation, particularly affecting cell proliferation and apoptosis [55, 56]. Research conducted in clinical settings has provided substantial evidence indicating that the disruption of homeostasis within circadian rhythms could potentially serve as an autonomous contributing factor to the development of cancer in humans [57-76] (Table 1). Filipski et al. [34] designed a two-stage carcinogenic model in which mice were inoculated with implants of Glasgow osteosarcoma or pancreatic adenocarcinoma tumors at varying day and nighttime points. It was established that distinct time points showed significant variations in the tumor formation rate, number, and volume of tumor foci. This suggests that the disruption of the circadian rhythm in mice is related to the accelerated growth of two types of malignant tumors, indicating that the host biological clock may play an important role in the endogenous control of tumor progression.

**Table 1:** Dysregulation of clock genes in human cancers.

Cancer type	Deregulated clock genes	References
**Breast cancer**	*Bmal1, Clock, Tim, Cry1, Per1, Cry2, Per2, Per3, Npas2*	[73-80]
**Ovarian cancer**	*Clock, Bmal1, Cry1, Cry2, Per1, Per2, Per3, CK1ε*	[51, 64, 81-84]
**Lung cancer**	*Clock, Per1, Per2, Per3*	[85-90]
**Pancreatic cancer**	*Bmal1, Cry1, Cry2, Per1, Per2, Per3, CK1ε, Clock, Dec1, Tim*	[91-93]
**Prostate cancer**	*Clock, Bmal1, Cry1, Cry2, Per1, Per2, Per3, Npas2, CK1ε*	[58, 66, 90, 94-97]
**Colorectal cancer**	*Per1, Per2, Per3, Clock, Npas2, Tim*	[53, 57, 90, 98-101]
**Endometrial cancer**	*Cry1, Per1, Per2, Per3*	[102-104]
**Non-Hodgkin’s lymphoma (NHL)**	*Cry2, Npas2, Bmal1*	[59, 105, 106]
**Osteosarcoma**	*Per2, CK1ε*	[65, 72]
**Leukemia**	*Bmal1, Clock, Cry1, Cry2, Per1, Per2, Per3, CKIε*	[107-111]
**Head and neck squamous cell carcinoma (HNSCC)**	*Bmal1, Cry1, Cry2, Per1, Per2, Per3, Tim, CKIε*	[112-114]
**Hepatocellular carcinoma**	*Cry2, Per1, Per3, Per2, Tim*	[68, 115]
**Diffuse large B-cell lymphoma**	*Clock, Bmal1*	[116]
**Glioma**	*Cry1, Cry2, Per1, Per2, Per3*	[105]
**Esophageal cancer**	*Dec1, Dec2, Per, Cry*	[117]
**Malignant pleural mesothelioma**	*Bmal1, Cry2, Per1, Per3, Npas2, Rev-erbα, Rev-erbβ, Tim*	[70-72]

### 3.1 The dysregulation of Clock genes in tumorigenesis

Clock genes play a crucial role in regulating the expression of proto-oncogenes, tumor suppressor genes, and transcription factors, thereby influencing tumorigenesis, development, and inhibition. Mutations in clock genes can lead to the upregulation of cell cycle suppressor genes and reduce the response of mutant cells to mitotic signals, significantly inhibiting cell growth and proliferation [81,118-120, 121]. Decreased expression of clock genes results in heightened expression of the proto-oncogene c-MYC, which drives the uptake of glutamine and arginine. The upregulation of c-MYC promotes anabolic processes, increases cellular proliferation, and alters cellular metabolism, contributing to tumorigenesis [122-124].

Epigenetic inactivation of *Bmal1* plays a key role in the development of hematological malignancies, such as non-Hodgkin's lymphoma and acute lymphoblastic leukemia. Specifically, *Bmal1* inactivation occurs through hypermethylation of the cytosine-phosphate-guanine (CpG) island promoter, leading to disruption of the circadian rhythm of specific target genes, including *c-MYC*, *catalase*, and *p300*, by interfering with the biological clock [110]. Inhibition of lung cancer metastasis and progression is elicited by *Per2* and *Bmal1* [125]. Methylation at the CpG site of *hPer3* was observed in patients with chronic myeloid leukemia [116].

The promotion of cancer cell growth *in vitro* and the enhancement of time-dependent tumor growth *in vivo* occur exclusively during specific periods of the day, as a result of the downregulation of either *Per1* or *Per2* [126]. Both *Per1* and *Per2* can inhibit the occurrence of breast cancer by promoting apoptosis *in vivo* and reduce the risk of tumorigenesis by indirectly inhibiting *c-MYC* transcription [127-132]. The tumor inhibitory function of *Per1* is evidenced by its ability to significantly impede the proliferation and growth of breast cancer cells, as demonstrated through a distinct diurnal expression pattern [91, 133]. *Per1* mediates tumor necrosis factor-α (TNF-α) to inhibit the proliferation of human pancreatic cancer cells (MIA PaCa-2) [134, 135]. In a study of sporadic and familial primary breast cancer, Winter et al. [78] found that the expression of *Per1* and *Per2* in tumor tissues was significantly lower than that in normal breast tissues, especially the expression of *Per1* in familial primary breast cancer, which was significantly lower than that in sporadic breast cancer specimens, indicating that the disorder of clock gene expression may be an important factor in the pathogenesis of familial breast cancer [77]. Zhao et al.’s [136] clinical study on 246 gastric cancer patients aged from 23 to 79 years old revealed a significant correlation between the expression of *Per1* and *Per2* and pathological stage. Notably, higher pathological stages were associated with lower expressions of *Per1* and *Per2*. Moreover, patients with low expressions of *Per1* and *Per2* had significantly shorter survival times than those with high expressions. Emerging evidence suggests that circadian genes may impact tumorigenesis through mechanisms beyond their traditional roles in regulating the molecular clock [137, 138]. While circadian rhythms orchestrate various physiological processes, circadian genes such as Per1 and Per2 have been implicated in cell cycle regulation, DNA damage response, and apoptosis, which are critical in cancer development and progression [138]. Several studies have demonstrated that Per1 and Per2 can have distinct effects on tumorigenesis. For instance, Per1 has been shown to enhance DNA damage repair and apoptosis, thereby exerting tumor suppressive effects [139]. Conversely, Per2 mutations are associated with an increased risk of certain cancers, suggesting a complex role in tumorigenesis that might be context-dependent [140].

The genetic variation in functional polymorphisms of the circadian genes *Cry2* and Ala394Thr in Neuronal PAS domain protein 2 (NPAS2) increases genetic susceptibility to non-Hodgkin's lymphoma [97, 111]. *Cry2* can promote ubiquitination and degradation of *c-MYC*, whereas deletion of *Cry2* leads to enhancement of *MYC*-driven lymphoma in mice [141]. High expression of *Clock*, *Per*, and *Cry* is associated with longer metastasis-free survival, and loss of co-expression of *Per3* and *Cry2* is associated with increased metastatic risk of breast cancer [142]. The development of endometrial carcinoma may be linked to disruptions in the biological clock resulting from CpG methylation of the promoter regions of *Per1*, *Per2*, or *Cry1* [108]. The relationship between clinicopathological parameters and the expression levels of *Cry1* and *Bmal1* core clock genes in epithelial ovarian cancer has been established. The independent prognostic factor of low expression of these genes has been identified, alongside staging and tissue subtypes [104]. Yang et al. [143] found that in 30 HCC samples, the mRNA levels of *Per1*, *Per2*, *Per3*, and *Cry2* were notably lower compared to the para-tumor tissues. Conversely, no significant changes were observed in the expression of CLOCK-BMAL1, *Cry1*, and CK1ε/δ. These findings indicate a lower presence of *Per1*, *Per2*, *Per3* and *Cry2* in HCC samples. This trend of decreased gene expression was also observed in colorectal and pancreatic cancer. In animal studies, chronic jet lag in mice increased their susceptibility to various cancers. Additionally, mice with mutations in clock genes were found to have a heightened risk of carcinogenesis. These animal model findings underscore the potential impact of circadian disruption on cancer development, paralleling the gene expression trends observed in human cancers [124, 113, 144].

A large-scale case-control study conducted within the general population yielded compelling evidence linking genetic variability in genes related to circadian rhythm with the incidence of prostate cancer [97]. Melatonin demonstrates upregulation of mRNA levels of *Clock*, and *Per2*, and downregulation of *Bmal1* mRNA, thereby suggesting a protective effect on rhythm loss during tumor progression [81, 145, 93]. The fundamental mechanism that propels the process of mesothelioma is the marked imbroglio of the clock gene [146].

**Figure 2.**
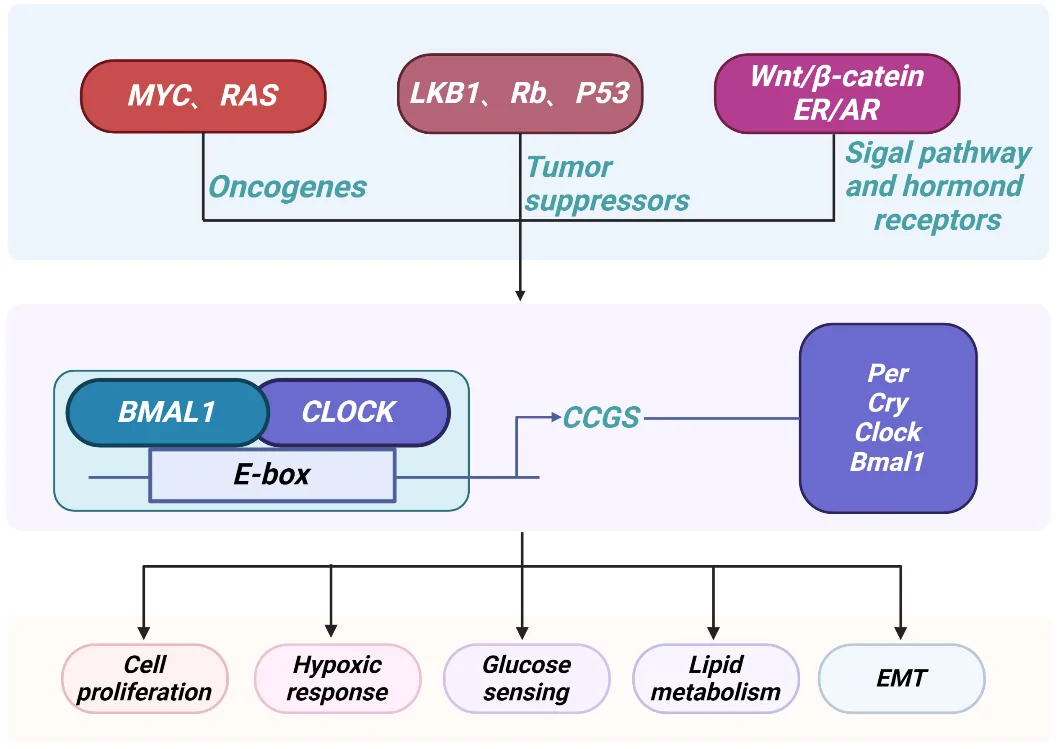
**Crosstalk of oncogenes and tumor suppressors with the circadian clock**. An overview of the input pathways associated with oncogenes and tumor suppressors, which have been identified as potential disruptors of the circadian clock. The resulting crosstalk between circadian system and these genes may bring about modifications in gene expression that impact critical metabolic pathways like glucose sensing, lipid metabolism, cell cycle and proliferation control, hypoxic response modulation, and regulation of the epithelial to mesenchymal transition (EMT). Grey depicts nuclear hormone receptors and transcriptional coregulatory proteins, purple represents transcription factors that interfere with CLOCK:BMAL1 transcription through enhancer box (E-box) binding, and green indicates tumor suppressors. CCGs: clock-controlled genes.

### 3.2 Correlation between oncogenes, tumor suppressors, and CRD(Fig. 2)

#### 3.2.1*MYC*

The MYC transcription factor is widely utilized for regulating cellular differentiation and proliferation by implementing a range of mechanisms, including the transcriptional amplification of target genes [147]. As previously discussed, the expression of biological clock genes has been observed to impact *MYC*, just as the regulation of C-*MYC* occurs through F-box and WD repeat domain containing protein 7 (FBXW7). The interplay between CRY2 and F-box and leucine-rich repeat protein 3 (FBXL3) stimulates the process of ubiquitination and consequent degradation of *C-MYC* [141, 148]. According to current reports, the circadian rhythm inhibitor CRY2 has been found to facilitate degradation of the oncogenic transcription factor MYC through E3 ligase complex FBXL3. Conversely, research has demonstrated that the removal of CRY2 results in an exacerbation of *MYC*-mediated lymphoma development in murine models [141]. It is of note that DNA damage not only intensified the interplay between FBXL3 and CRY2 [149], but a recent investigation discovered that FBXL3 was the singular F-box substrate to elicit a response to DNA damage among the four, manifesting a notable augmentation in the recruitment of CUL1 [150]. In addition, CLOCK/BMAL1 is directly bound to the promoter region of *C-MYC*, resulting in its downregulation. Conversely, MYC was found to hinder CLOCK/BMAL1-dependent transactivation of PER1 expression [151]. These conclusions are bolstered by the adverse relationship between BMAL1 expression and MYC in a sample of 102 human lymphomas [152]. Furthermore, *Per2* mutant mice (*Per2^m/m^*) are susceptible to lymphoma and exhibit upregulation of *C-MYC* expression and its target gene *CCND1* [153]. Taken together, these data suggest an opposing relationship between MYC and the circadian transcriptional axis that controls cell survival and proliferation pathways.

#### 3.2.2 RAS

The *RAS* gene family (*K-RAS*, *H-RAS* and *N-RAS*) is encoded by different genes located on each of the three chromosomes [154]. RAS proteins are significant constituents of signaling pathways involved in cellular growth and proliferation. In certain cases, mutations in *RAS* can result in anomalous cell proliferation, eventually leading to tumorigenesis [155, 156] Mutations in RAS can accelerate changes in cellular metabolism and influence circadian rhythms. Recent research has uncovered that while wild-type RAS interacts with circadian rhythms, mutant RAS may significantly impact these rhythms [157, 158]. The study conducted unveils that human keratin-forming cells, which were *H-RAS* transformed, manifested a lack of gene expression for *Per2* and *Bmal1*. Conversely, the expression of *Cry1* and *Clock* was upregulated within the synchronized cells [159]. The introduction of *H-RAS* or *K-RAS* results in a notable extension in the duration of the circadian cycle. Such an occurrence infers that transformation, which depends on *RAS*, hinders the expression of circadian genes [159]. Notably, cells exhibiting *Per2* mutations or *Cry1/2* deletions have shown susceptibility to transformation by *H-RAS.* Conversely, mouse embryonic fibroblasts (MEF) displaying *Bmal1* deletions or *clock* mutations have demonstrated resistance to oncogene-induced transformation [160]. This RAS-mediated transformation relies on the activation of transcription factor 4 (ATF4) to suppress the activation of cell cycle regulators and tumor suppressor genes p16INK4a and p19ARF [161]. Utilizing a genetically engineered mouse model (GEMM), it was observed that CRDs expedited tumor development in cases where tumors manifested themselves gradually over a course of several months [125]. The RAS signaling pathway and its downstream mitogen-activated protein kinase (MAPK) pathway were found to regulate circadian rhythms in Drosophila animal experiments, and the MAPK pathway is governed by circadian oscillations [162]. Subsequent research indicated that the extracellular regulated kinase (ERK) pathway within the MAPK pathway possesses the capability to directly phosphorylate CLOCK, an action that serves to bolster the expression of biological clock genes [163]. Glycogen synthase kinase-3 (GSK-3) is a regulator of CRY, and PER and can control the stability of CLOCK [164]. When GSK-3 is inhibited, it stabilizes the CLOCK protein. Conversely, activation of GSK-3 leads to the degradation of the CLOCK protein [165, 166]. Spengler et al. [167] found that the inhibition of GSK-3 by RAS results in a reduction in clock degradation through phosphate degradation sites, ultimately leading to an increase in transcription that is clock dependent. These findings indicate that disruptions to the molecular processes governing circadian rhythms may yield varying outcomes on *RAS*-mediated transformation, potentially in a tumor-dependent manner. Thus, distinct cell types may exhibit unique responses to oncogene-driven transformation *in vivo* and *in vitro*.

#### 3.2.3 Liver Kinase B1(LKB1)

AMPK is a highly conserved protein in mammalian cells that functions as a key regulator of metabolic and energy homeostasis [168]. LKB1 protein is an upstream kinase of AMPK. It has been observed that mutations or deletions of LKB1 are prevalent in malignant cells, particularly in non-small-cell lung cancer, with a prevalence of up to 35% [169]. Recent studies have shown that the tumor suppressor function of LKB1 is dependent on the activation of four subfamilies of AMPK-related kinases, which specifically include microtubule affinity-regulated kinases (Mark1-4) [170, 171]. AMPK phosphorylates CRY1 on two serines (S71 and S280), thereby disrupting its interaction with PER2 [172], while promoting the binding of CRY1 to FBXL3, leading to CRY1 ubiquitination and degradation [173]. It is anticipated that all kinases activated by LKB1 would phosphorylate substrates exhibiting amino acid sequences similar to those phosphorylated by AMPK. However, the potential of MARKs to phosphorylate CRY1 or CRY2 remains unclear. Further research is needed to elucidate this potential and understand its implications. Recent research has revealed that the nuclear localization and activity of AMPK in mouse liver bears an inverse relationship with the intranuclear protein abundance of CRY1. Activation of AMPK results in the phosphorylation of CRY1, thereby affecting circadian rhythms [173, 174]. Furthermore, AMPK has the capability to modulate the mRNA content of nicotinamide phosphoribosyltransferase (NAMPT). In a study using a transgenic mouse model, it was demonstrated that the level of NAMPT in skeletal muscle increases with the activation of AMPK during metabolic stress. Ultimately, the activation of NAD through the elevation of NAMPT levels by AMPK results in the regulation of circadian rhythms. This regulatory process occurs by activating silent information regulator (SIRT) activity, leading to increased control over circadian rhythms [175]. Metformin effectively reduces blood glucose levels through the activation of AMPK. Recent findings suggest that mice treated with metformin experience a shortened circadian cycle, which is attributed to the facilitation of CK1ε/δ phosphorylation by AMPK. This ultimately promotes *mPer2* degradation and augments activity levels [176]. The current mechanism of the clinical application of metformin and antimetabolic therapy for antitumor treatment is to alter the biological clock by activating AMPK [177]. In summary, the reduction or deletion of LKB1 and AMPK alters circadian oscillations.

#### 3.2.4 RB

RB inactivation can cause retinoblastoma in children [178]. RB inhibits cell growth by preventing adenovirus early region 2 binding factor (E2F) from activating the expression of cell cycle-promoting genes [179]. Phosphorylation of RB by cyclin-dependent kinases 4 and 6 (CDK4/6) prevents its interaction with E2F, and CDK4/6 inhibitors are effective in treating tumors with intact RB function [179]. Treatment of simulated human osteosarcoma cells (U-2OS) with chronic CRD enhances phosphorylation of CDK4/6 protein and RB [180]. The occurrence of this phenomenon can be effectively thwarted with the genetic deletions of *Bmal1*, *Cry1*, and *Cry2*. Our latest research has indicated that both *Cry1* and *Cry2* exhibit interactions with various E2F family transcription factors, although only *Cry2* actually promotes the ubiquitination and subsequent degradation of *C-MYC* [181]. Study found that both CRY1 and CRY2 can interact with E2F family members and stimulate their interactions with FBXL3 [141]. The maintenance of stable E2F4 and E2F8 levels is highly dependent on the regulation of *Cry1* and/or *Cry2* expression. However, the regulation of cryptochromes has less influence on the levels of E2F1 protein [182]. *Cry1* and *Cry2* may affect E2F-dependent transcription through direct repression, but this requires further study.

#### 3.2.5 P53

*P53* is a prominent oncogene, with its coding product serving as a nuclear transcription factor [183]. A common observation in tumor cells is the presence of *p53* mutations or deletions which lead to aberrant metabolism, compromised cell cycle regulation, and suppressed apoptosis [184]. It was found that *p53* expression has a circadian rhythm [185, 186]. The biological clock is regulated by *p53* and *Per2* expression is transcriptionally controlled by *p53* [187]. The *p53* response element overlaps with the E-box sequence in the *Per2* promoter, such that *p53* occupancy blocks CLOCK:BMAL1 promoter recruitment [187]. PER2 forms a stable complex with P53, thereby preventing MDM2-mediated ubiquitination and proteasomal degradation of P53 [188]. Clock proteins play a crucial role in regulating the stability of P53 at the cellular level, resulting in the direct control of *p53*. Furthermore, the discovery that *p53* nuclear shuttling is induced through ectopic expression of *Per2* supports this assertion [189]. Inactivation of *Per2* by mutation causes delayed accumulation of *p53* in tumor-bearing mice exposed to ionizing radiation, and inhibition of *p53* activation leads to uncontrolled continued cell proliferation [153]. Recent findings provide additional support for the notion that PER2 stability is governed by an unconventional circadian pathway whereby MDM2, a ubiquitin ligase, directly targets PER2 [190]. Hua et al [79] revealed that elevated expression of *p53* resulted in modifications of apoptosis-related genes in mice afflicted with lung cancer. Additionally, heightened mRNA and protein levels of *p53* were detected in Lewis lung carcinoma (LLC) cell lines with high *Per2* expression, compared to control samples. Consequently, this study has established that augmenting the expression of *mPer2* can effectively impede tumor cell growth and instigate apoptosis in lung cancer-afflicted mice tumor cells, through regulation of the mitochondrial signaling pathway by *p53*. Miki et al [187] found that within the suprachiasmatic nucleus (SCN), *p53* binds to the *Per2* promoter, preventing binding of the *Per2* promoter and CLOCK/BMAL1, resulting in a suppression of *Per2* expression. Moreover, it was discovered that varying levels of *p53* cause a phase shift in the physiological behavior of mice. It is deduced that *p53* has the ability to influence the expression of the *Per2* gene and its protein product, and thus, impact circadian rhythms by regulating *Per2*. Two complementary research studies verified that the PER2 protein in the cytoplasm can form a dimer with P53, thereby stabilizing P53 and facilitating its entry into the nucleus, both under normal and genotoxic stress conditions [188, 191]. Thus, there is a bidirectional regulation between *p53* and *Per2* [93]. Recent research has indicated that the downregulation of the *Bmal1* gene in pancreatic cancer cells stimulates cell growth. Conversely, the over-expression of *Bmal1* hinders cell cycle and proliferation control in a *p53*-dependent fashion [192]. The data presented indicates an alternative function for clock proteins in the modulation of P53. The observed variations in clock-mediated P53 activity could be ascribed to the diverse roles of P53 in UV stress, DNA damage response, DNA repair, or cell type-specific apoptotic pathways [193-196].

### 3.4 Correlation between dysregulated signaling pathways in CRD and tumorigenesis

CRD is not only closely related to these common oncogenes and tumor suppressors genes, but also participates in crosstalk with some signaling pathways [20]. It has been reported that there is a two-way crosstalk between the biological clock mechanism and Wnt/β-catenin signaling, especially in colorectal cancer [197-199]. Enhancement of β-catenin signaling through interaction with an F-box protein of the Skp1-cullin 1-F-box (SCF) ubiquitin E3 ligase family called β-TrCP destroys the stability of PER2 protein levels in the intestinal mucosa [200]. The present studies highlight a bidirectional communication between the β-catenin signal and the diurnal molecular mechanism in the gut. The involvement of clocks in Wnt/β-catenin signal-dependent cancers remains an open question. Notably, Wnt signaling plays a critical role in cellular differentiation and tissue formation [201, 202]. Through the utilization of a three-dimensional *in vitro* organ model to examine ordinary intestinal stem cells, it was discovered that the cellular circadian clock effectively obstructed the advancement of mouse epithelial cell cycles via its dependence on the secretion of Wnt [203]. In addition, the destruction of the biological clock can lead to the division of normal intestinal stem cells in drosophila melanogaster [204]. As a result, disturbing the biological clock can lead to upregulation of Wnt signaling, thereby altering the control of intestinal stem cell proliferation. Although the biological clock is related to cancer stem cells, the exact role and detailed molecular mechanism of the biological clock in different types of cancer are unclear [205, 206]. Estrogen receptor α (ER α) is closely related to hormone-dependent breast cancer and the biological clock [207]. The CLOCK protein interacts with ER α, whose activity is potentiated by estrogen. This hormone stimulates the overall number of clocks, leading to an increase in clock-dependent transcriptional activity, enhancing ERα-mediated transcription, and ultimately promoting proliferation in breast cancer cell lines (MCF7 and T47D) [208]. Intriguingly, PER2 has been reported to modulate the stability of ER α protein levels, and estrogen is known to trigger PER2 expression, which suggests a regulated feedback mechanism [94]. In addition, PER1 has been reported to inhibit the transactivation of androgen receptor (AR) through direct interaction, and the ectopic expression of PER1 in human prostate cancer cells (LNCaP) reduces the expression of known AR target genes [94]. While the results obtained are noteworthy, there is still ambiguity regarding the interplay between the biological clock and nuclear hormone receptors within the human body. Specifically, the influence of progesterone receptor circadian rhythm in regulating breast cancer requires further evaluation. Furthermore, although thyroid hormone secretion remains consistent throughout the diurnal cycle, the oscillation of thyroid hormone receptor α and β expression remains dynamic. As such, additional inquiry is required to shed light on these internal associations.

## 4. CRD and tumor metabolism

**Figure 3.**
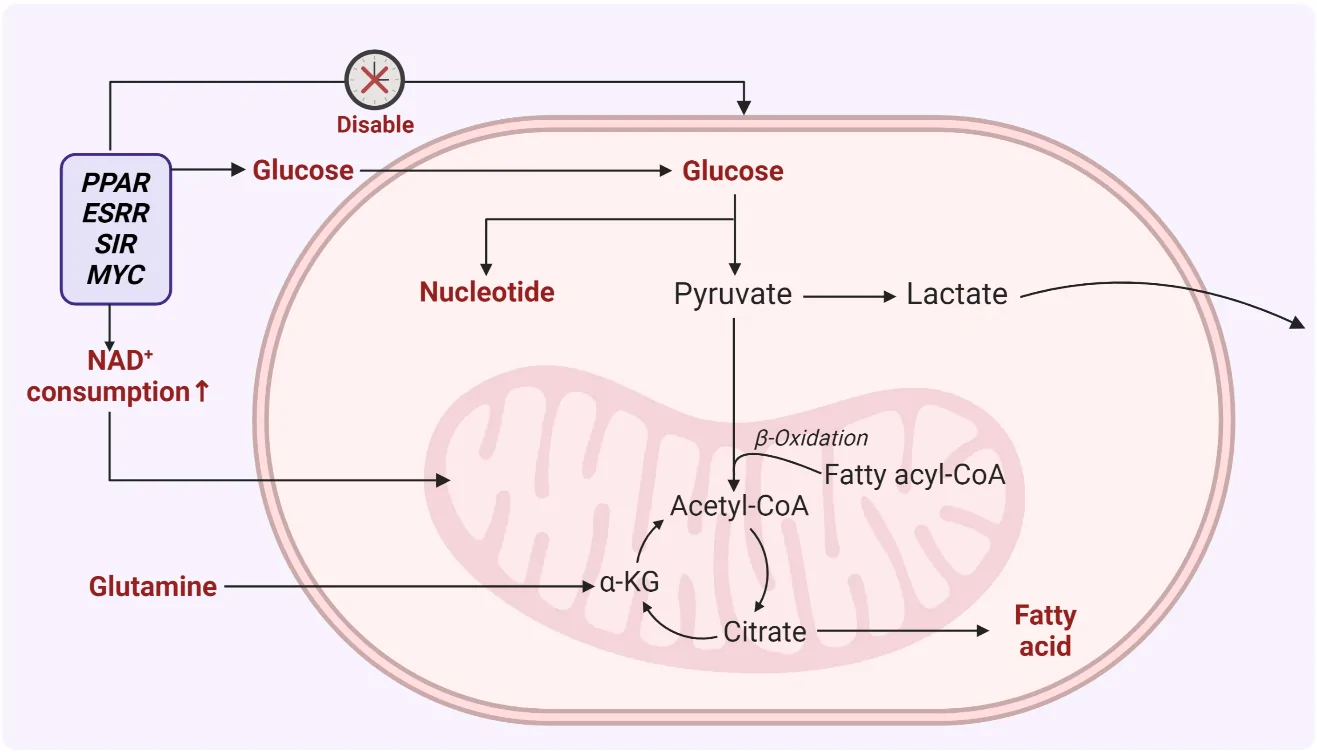
**Regulation of cell proliferation via metabolic processes by both clock and non-clock TFs**. In cells with robust proliferation, non-clock transcription factors such as PAPR and MYC impede the clock system while stimulating reductive metabolism, resulting in NAD^+^ consumption. Consequently, the cellular metabolism shifts towards aerobic glycolysis and glutamine oxidation, as well as nucleotide and lipid synthesis. αKG, α-ketoglutarate

Recent research suggests that the biological clock plays a critical role in regulating cell metabolism through chromatin remodeling [2, 209-211]. Clock-mutant mice are susceptible to obesity and exhibit symptoms of metabolic syndrome, which may include elevated blood lipids, hyperglycemia, and liver steatosis [212]. The strong correlation between the biological clock and metabolism is underscored by these findings. While a comprehensive understanding of how clock proteins regulate metabolism remains elusive, prevailing evidence suggests that chromatin remodeling plays a critical role. Consequently, some clock regulators may be influential at the intersection between epigenetics and metabolism [213, 214] (Fig. 3).

### 4.1 The role of CRD in cancer metabolic reprogramming

Cancer cells exhibit a vast spectrum of metabolic irregularities [215]. The most prominent metabolic characteristics of cancer cells are aerobic glycolysis (Warburg effect), glutamine oxidation, lipogenesis and increased nucleotide synthesis [216]. Mouse embryonic fibroblasts isolated from *Bmal1^-/-^* mice showed high glycolysis, increased lactic acid production, and decreased lipid oxidation and ATP production, similar to the metabolic characteristics of cancer cells [217]. It is noted that a clock-mediated glycolysis state similar to that seen in tumors can potentially be achieved through transcriptional regulation of genes that encode glycolytic enzymes including pyruvate dehydrogenase kinase 1 (PDK1) and lactate dehydrogenase A (LDHA) [217]. A number of genes, whose protein products play vital roles in metabolic processes, demonstrate a circadian rhythm in their expression. These encompass glucose-6-phosphatase and PCK2, which are involved in gluconeogenesis, pyruvate kinase, which is responsible for glycolysis, glucokinase, involved in glycogen synthesis, glucose transporter 2, which facilitates glucose transport, and HMG-CoA reductase, which participates in cholesterol metabolism [218]. A recent investigation has revealed that malignant cells exhibiting an elevated CRDscore are distinguished by the activation of crucial metabolic pathway glycolysis, as well as the epithelial-mesenchymal transition pathways [219]. Meanwhile, the ongoing development of biomarkers linked to circadian rhythms holds considerable promise in the realm of prognosticating tumor outcomes and assessing the effectiveness of immunotherapy [220]. Recent research has discovered that the absence of BMAL1 has a disruptive effect on the synchronization of biological rhythms and amplifies the manifestation of fibrotic characteristics in various types of tumors. As a consequence, this abnormality triggers the proliferation of cancer-associated fibroblasts (CAFs) [215]. CAFs are responsible for inducing a series of metabolic alterations, ultimately fostering the spread of tumors and tumor immune evasion [221]. The acceleration of Apc-driven colorectal cancer (CRC) pathogenesis *in vivo* was observed through the utilization of an intestinal organoid model. It was found that CRD is responsible for this acceleration. The APC-mediated hyperactivation of Wnt signaling was identified as a key factor in this process. This hyperactivation leads to the up-regulation of *c-Myc*, a well-known target of Wnt signaling, which subsequently drives an increase in glycolytic metabolism [222]. Recent studies have provided evidence suggesting that CRD is linked to a heightened susceptibility to obesity and obesity-related ailments [223-225, 226]. It has been established that perturbations in the circadian rhythm play a pivotal role in tumor development and are correlated with an increased occurrence and progression of various cancers, including breast, prostate, colorectal, and thyroid cancer [227].

**Figure 4.**
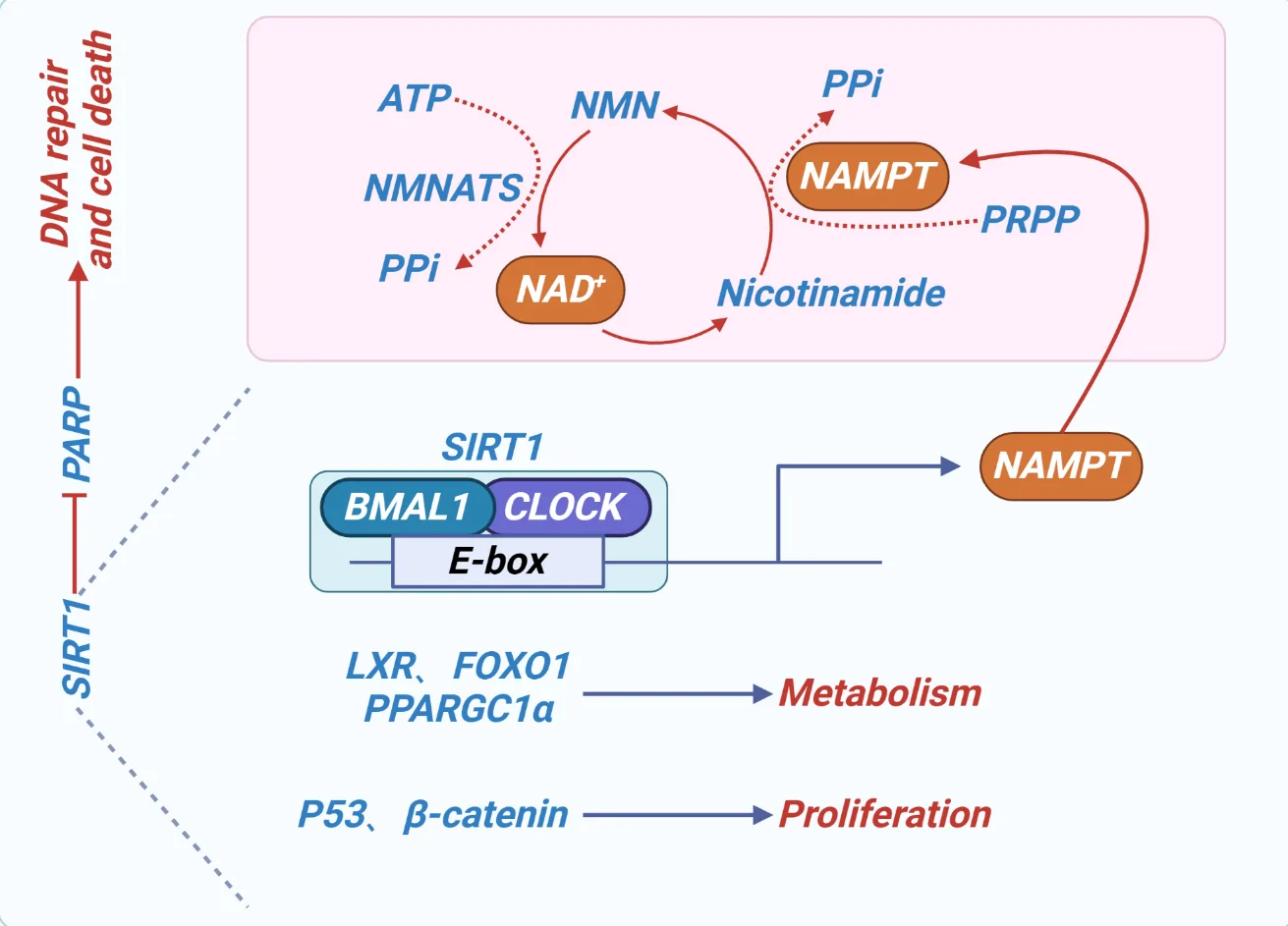
**Regulation of the NAD^+^ salvage pathway by circadian rhythm**. The expression of NAMPT is governed by the circadian clock, which controls the rate-limiting enzyme involved in biosynthesis of NAD^+^ from nicotinamide in mammals. NAMPT facilitates transfer of a phosphoribosyl residue from 5-phosphoribosyl-1- pyrophosphate (PRPP) to nicotinamide, resulting in the production of nicotinamide mononucleotide (NMN) that is further converted to NAD^+^ by nicotinamide mononucleotide adenylyltransferases (NMNATs). Oscillations in NAMPT levels lead to circadian variations in NAD+ levels that ultimately determine the activity of SIRT1 and PARPs. Consequently, SIRT1 governs the oscillatory levels of its own coenzyme, NAD^+^, while also regulating proteins involved in metabolism and cell proliferation through deacetylation. Orange indicates circadian oscillation.

The expression of several nuclear receptors, including peroxisome proliferator-activated receptor family members (PPAR α, PPAR γ and PPAR δ) and estrogen related receptor family members (ESRR α, ESRR β and ESRR γ), is specific to different tissues [228]. PPAR γ coactivator 1 α (PPARGc1 α), which follows a circadian expression pattern in metabolic tissue, plays a crucial role in exercise activity, body temperature regulation, and metabolic rates, and its absence in mice can lead to defects in these areas [229]. Disruptions in regulatory factors such as SIRT1, an essential deacetylase involved in various biological activities, can result in metabolic pathway defects, proliferation, and ultimately cancer. SIRT1 plays a significant role in metabolism by deacetylation of several proteins and histone deacetylation to regulate gene expression [230]. SIRT1 regulates tumor-related metabolism by deacetylating LXR and FOXO1, which affects lipid metabolism and insulin sensitivity. This regulatory mechanism significantly influences cell proliferation and survival in cancer cells [231-233]. One of the attractive features of SIRT1 is that it depends on the enzyme activity of NAD^+^ [234]. The diurnal oscillation of SIRT1 activity showed that the intracellular NAD^+^ level could also oscillate (Fig. 4). A study showed that NAD^+^ levels show bimodal oscillations in the mouse liver [235]. The biological clock controls the expression of NAMPT, which is the key rate-limiting enzyme in the remedial synthesis pathway of NAD^+^ [235, 236]. Intriguingly, another NAD^+^-dependent enzyme, poly (ADP-ribose) polymerase (PARPs), has been shown to interact functionally with SIRT1 [237]. Given the direct control of SIRT1 deacetylase activity by NAD^+^, circadian regulation of NAD^+^ levels appear to be an important regulatory mechanism controlling circadian rhythms, metabolism and cell growth. Intriguingly, alterations in NAMPT levels are associated with metabolic disorders and cancer [238], while FK866 (a highly specific NAMPT inhibitor that abolishes NAD^+^ circadian oscillations and SIRT1 cycle activity) can induce apoptosis in human cancer cells [236, 239]. However, further studies are needed to reveal the exact role of circadian rhythm control of SIRT1 activity in the regulation of metabolism and tumorigenesis.

### 4.2 Crosstalk between hormones and the circadian clock

Certain genes demonstrate a circadian expression pattern and the proteins resulting from them assume pivotal functions in metabolic processes such as glucokinase, glucose-6-phosphatase, pyruvate kinase, glucose transporter 2, and HMG-CoA reductase. As the SCN regulates daily fluctuations in hormone production and secretion, specifically melatonin and corticosteroids, disturbances in the circadian rhythm cause corresponding changes in their circulating levels [240]. The control of melatonin levels is governed by central pacemakers in the SCN, which operate through polysynaptic pathways. This hormone is inhibited by light [241]. One of the roles of melatonin is to regulate glucose homeostasis, which in turn prevents cell and tumor growth [242-244]. The literature indicates that prolonged exposure to low light conditions is an apparent contributing factor in the growth of liver cancer and the synthesis of mammary epithelial cells. This is attributed to the inhibition of melatonin synthesis [245, 246]. Various epidemiological investigations have shown a consistent correlation between high melatonin levels and a lower incidence of cancer. These findings provide credible evidence supporting the potential role of melatonin in suppressing tumor growth [247, 248]. Aligned with this finding, the administration of melatonin has been shown to decrease estrogen levels and impede DNA synthesis in breast tissue [249]. Disruption of the circadian rhythm of glucocorticoid levels is believed to be a potential factor in the development of tumors. The production of adrenal glucocorticoid is primarily controlled by the hypothalamic-pituitary-adrenal axis, while intrinsic clocks located within the adrenal gland and the sympathetic nervous system also regulate glucocorticoid levels [250-253]. When the SCN is disrupted, the circadian rhythm of glucocorticoid levels becomes disturbed, potentially harming clock oscillations and increasing the risk of tumorigenesis [254]. Additionally, epidemiological studies have shown that glucocorticoids may play a part in stress-related cancer progression [255]. From a mechanistic standpoint, the transcriptional regulator TSC22D3 appears to be a key player in impeding treatment-induced anticancer immunity when stress-induced increases in glucocorticoids occur. Changes in glucocorticoid levels may also affect lymphocyte function, thereby impacting antitumor immunity [256, 257]. Moreover, cryptochromes, which evolved from bacterial light-activated DNA repair enzymes [258], have been shown to contribute to various cellular functions that may influence cancer growth, in addition to generating circadian rhythm [182].

## 5. CRD and tumor immune response

**Figure 5.**
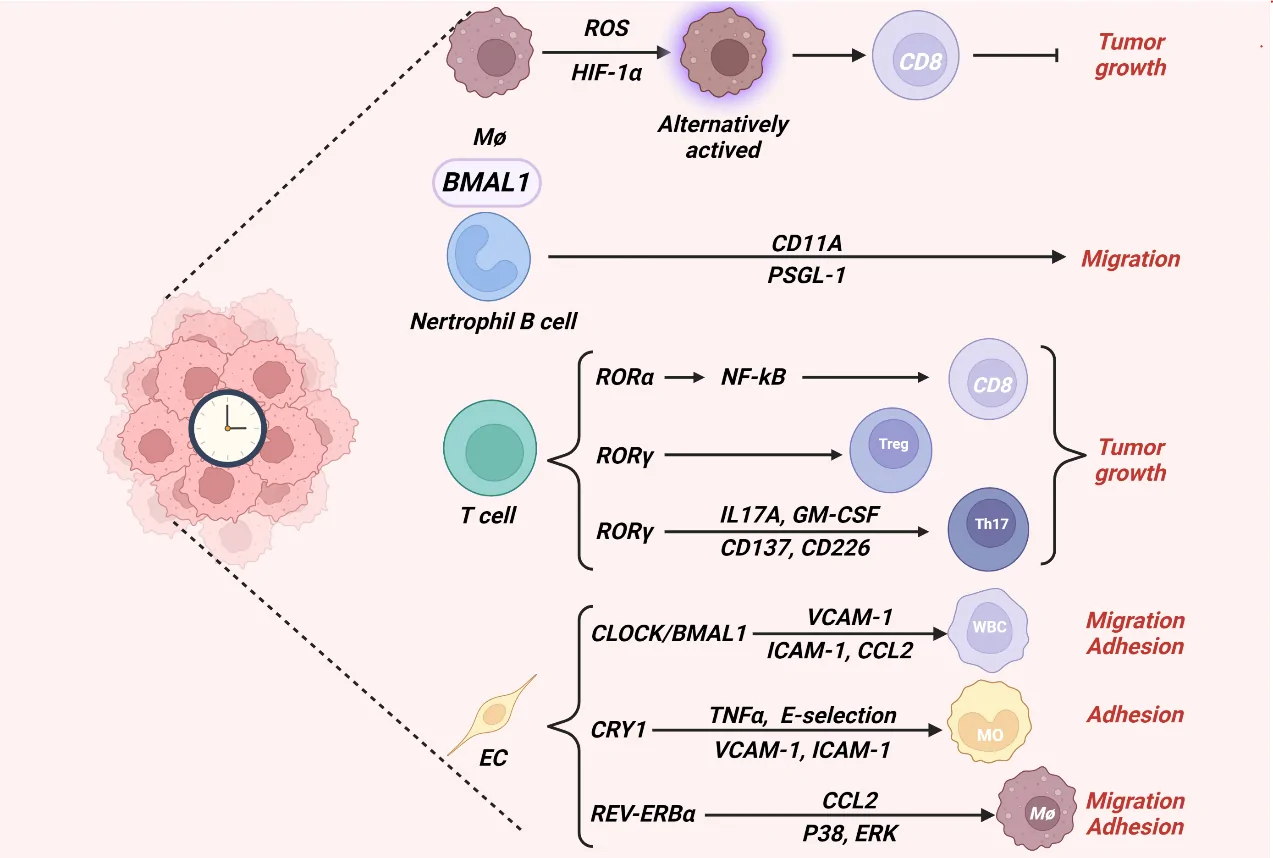
**Clock components that affect immune response**. BMAL1 inhibits the production of reactive oxygen species (ROS) and hypoxia-inducible factor 1α (HIF1α) in macrophages (MΦ). Moreover, BMAL1 has been shown to have a significant impact on macrophage alternative polarization and CD8^+^T cell-mediated immune response, thereby influencing tumor growth. In the case of B cells and neutrophils, BMAL1 has been found to regulate their migration ability by modulating the expression of promigratory molecules. Further, the differentiation and activation of T cells, such as CD4^+^Th17 cells, CD8^+^Tc cells, and Tregs, can be modulated by RORγ and RORα through regulation of specific factors and pathways that have implications for tumor growth and the antitumor immune response. The regulatory influence of EC clocks such as CLOCK, BMAL1, REV-ERBα, and CRY1 can impact the adhesion and migration of white blood cells (WBC), monocytes (Mo), and macrophages by modulating the expression of promigratory molecules such as VCAM-1, ICAM-1, and E-selectin as well as cytokines such as CCL2 and TNF-α. Furthermore, the activation of ERK and P38 pathways can also potentially impact tumor growth. GM-CSF: granulocyte-macrophage colony-stimulating factor; IL-17A: interleukin 17A; Th17 cells: type 17 T helper cells; Tregs: regulatory T cells

The immune system comprises immune organs, immune cells, and immune active substances, serving immune surveillance, defense, and regulation functions [259]. The expression of circadian rhythm genes is widespread in most immune cells, resulting in diurnal changes in the functioning of both innate and adaptive immune cells [408, 260]. The rhythmic expression of immune cell clock genes has profound implications on immune cell function, including the generation of daily rhythms in cytokine and chemokine synthesis and release [260-263]. Recent studies have shed light on the involvement of biological rhythms in the regulation of the immune system [264-266] (Fig. 5).

It is worth noting that diurnal rhythms impact not only immune cells, but also levels of immune effector molecules [267]. Disruptions to the circadian clock can therefore lead to disruptions in immune rhythms, potentially impairing normal immune responses. Notably, disruption of the circadian rhythm has been linked to several dysfunctions in intestinal intraepithelial lymphocytes, including the promotion of regulatory B cell dysfunction and CD4^+^T cell apoptosis, ultimately leading to enteritis [268]. Abnormal biological rhythms can compromise the immune system and further facilitate tumor growth [15]. In rats subjected to a chronic shift model, disrupted biological rhythms led to rhythm disorder of cytolytic function in natural killer (NK) cells, as evidenced by the altered expression of NK cell clock genes *Per2* and *Bmal1*. This, in turn, promoted the growth of intravenously injected MADB106 tumor cells in the lung [269]. The innate immune system's macrophages also exhibit rhythmic oscillations that are regulated by the biological clock and play a critical role in tumor immunity [15]. CRD can contribute to tumorigenesis and development by hindering adaptive immunity. The CD4^+^/CD8^+^T cell ratio in the breast cancer tumors of mice with rhythm disorders increases, while the immunosuppressive CD4^+^FoxP3^+^Treg is notably enhanced, resulting in the elevation of the Treg/CD8^+^T cells ratio, creating an immunosuppressive microenvironment that advances the malignant progression of breast cancer [270].

Recent research demonstrates that the inner core rhythm genes of tumor cells are extensively dysregulated. Gene correlation analysis indicated that the immune checkpoints of tumor cells, such as programmed cell death 1 ligand 1 (PD-L1), PD-L2, PD-1, CTLA-4, and TGF-β1, display a positive correlation with the expression of core rhythm genes. Furthermore, ChIP-seq data in normal mouse liver indicates that the regulation of PD-L1 transcription is guided by biological rhythms [271]. There is a speculative notion that the rhythm disorder found in tumor cells has the potential to affect the expression of immune checkpoint molecules, which could facilitate tumor immune escape. However, further experimental evidence is required to substantiate this claim. Recent *in vivo* observations confirm the existence of cytokine secretion rhythms, indicating that when wild-type mice were transferred to the dark phase, the serum levels of IL-6, IL-12, CCL5, chemokine (C-X-C motif) ligand 1 (CXCL1), and C-C motif chemokine ligand 2 (CCL2) were all elevated after lipopolysaccharide (LPS)-induced stimulation [272]. An alternative explanation for the rhythmic production of cytokines may be linked to the regulation of glucocorticoids, which can suppress the production of inflammatory mediators in macrophages and are also subject to circadian rhythms. It is probable that the circadian clock plays a crucial role in regulating these oscillations [273, 274]. Gibbs et al. [275] discovered that mice with myeloid-specific Bmal1 deletion did not show any changes in the levels of IL-6 serum, induced by Lipopolysaccharide (LPS). The study further explored the potential association between REV-ERBα and the circadian and inflammatory pathways, where the expression of the regulatory factor REV-ERBαwas found to be temporally regulated in peritoneal macrophages, which was disrupted with Bmal1 depletion. Notably, REV-ERBα deficiency led to the cessation of rhythmic immune responses to LPS, indicating a potential interrelation between BMAL1, REV-ERBα, and IL-6 production in macrophages. Mice exhibiting a deficiency in *Per1* and *Per2* displayed heightened levels of IL-1β and TNF-α expression during both basal states and LPS stimulation [276]. Additionally, removal of the clock genes *Cry1* and *Cry2* resulted in a prolonged elevation in the expression of IL-6, IL-1β, and TNF-α [277].

In normal physiological circumstances, the immune system experiences daily oscillations in its composition and function, which is governed by the biological clock system. The system effectively combats invading pathogens, damaged cells, and tumor cells, thereby preserving the body's overall well-being. However, if the feedback mechanism of the circadian clock is compromised, the immune system may remain persistently suppressed, thus diminishing its ability to eliminate tumors effectively. In addition, the disruption of rhythm in tumor cells may facilitate their immune-evading mechanism. Thus, the disruption of immune regulation caused by rhythm disorder is a crucial factor in the emergence and progression of tumors.

## 6. The role of CRD in tumor therapy

### 6.1 Chronochemotherapy and CRD

The efficacy of drugs and their associated side effects are key considerations when implementing a chemotherapy regimen for cancer patients [17]. Chronochemotherapy, which entails adjusting drug administration in accordance with biological rhythms, can not only enhance drug efficacy but also mitigate adverse reactions [278-280]. Chronochemotherapy involves administering medication at the optimal phase of the circadian rhythm to achieve maximal efficacy. Studies conducted previously have found that the timing of medication is a contributing factor to the survival rate of children diagnosed with acute lymphoblastic leukemia. Specifically, administering 6-mercaptopurine and methotrexate at night resulted in a 5-year survival rate of 80%, whereas administering these drugs in the morning yielded a significantly lower rate of only 40% [281]. As a result, regular maintenance chemotherapy has been widely accepted as the preferred approach to treating acute lymphoblastic leukemia in children. The effectiveness of anticancer drugs is frequently hindered by their undesirable side effects and toxicity. Chronochemotherapy offers a solution to this issue by providing a means to reduce adverse effects. One promising approach involves the use of a newly developed oral fluoropyrimidine drug which is designed to be released after a predetermined delay, thereby reducing potential negative effects [11]. Another study entailed conducting an irinotecan pharmacokinetics evaluation and toxicity assessment on 31 cancer patients. The patients who received the drug through the second method of administration experienced less severe diarrhea during regular drug administration. This finding highlights the effectiveness of chronochemotherapy in reducing the adverse reactions of drugs [282]. Furthermore, chronochemotherapy shows promise in enhancing the survival rate and quality of life of cancer patients by mitigating the cytotoxic effects of anticancer drugs [283-285]. Various phase I to phase III clinical trials have also indicated the efficacy of chronochemotherapy in treating diverse types of tumors [286, 287]. From a mechanistic standpoint, certain anticancer medications have demonstrated heightened cytotoxicity toward cells during specific phases of cellular division. This indicates that optimizing dosing intervals based on circadian rhythm-related drug properties could potentially lead to favorable clinical outcomes for patients [288, 289]. While the liver is a well-known rhythmic organ, it has been observed that livers with tumor metastases do not exhibit a distinct circadian rhythm. However, administering anticancer drugs directly into the hepatic artery in a time-regulated manner has shown beneficial effects in patients with liver metastases [290, 291]. In an international clinical trial, this approach proved to be a safe and effective therapy [292].

However, findings from a small study [293] advocating for the effectiveness of chronotherapy in ovarian cancer were not replicated in subsequent larger studies [294, 295]. It is pertinent to note that a sizable European investigation [296] on the effects of chronochemotherapy on colorectal cancer did not yield any beneficial outcomes for the study population as a whole. It is worth mentioning that while chronochemotherapy showed favorable results for male patients compared to conventional treatment modalities, the utilization of chronotherapy by female patients resulted in a concerning 38% increase in mortality [297]. This suggests that chronochemotherapy may be harmful to specific populations and provides important experience for subsequent clinical trials of chronochemotherapy. It also suggests that the empirical use of chronochemotherapy in cancer treatment has not yielded sufficiently consistent results to justify routine clinical use. The real application of chronochemotherapy in cancer treatment is still in its infancy. More clinical trials are needed to expand its clinical application, particularly in optimizing treatment schedules, understanding patient-specific circadian rhythms, and investigating the effects of chronochemotherapy across different age groups and genders.

### 6.2 Targeting core elements of the biological clock in tumor therapy

**Figure 6.**
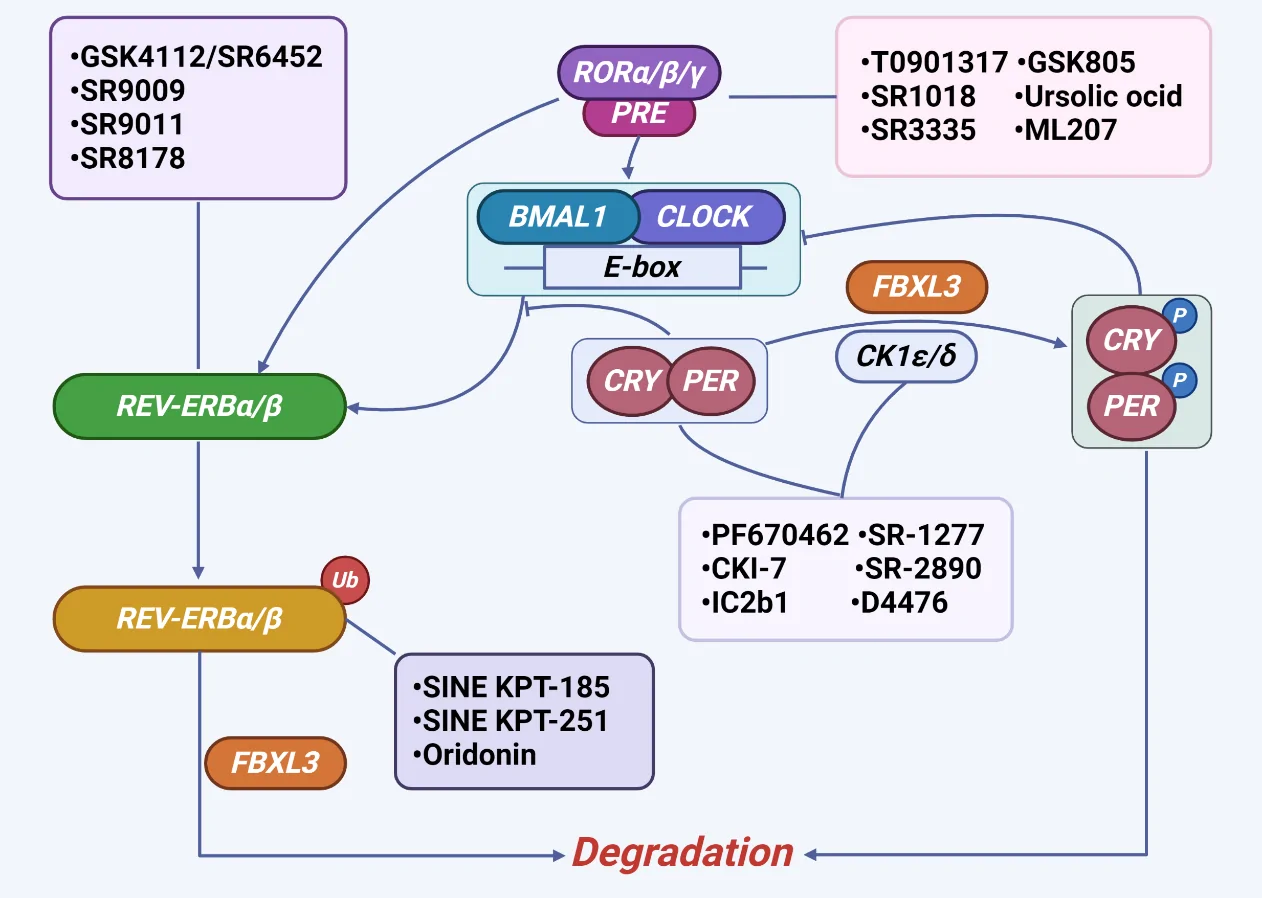
**Directly pharmacological targeting circadian clock for cancer therapy**. The focus on targeting circadian clock components has gained significant interest as a potential therapeutic strategy for cancer treatment. Multiple pharmacological agents have been identified that target various components of the circadian clock, such as REV-ERBα/β, RORα/β/γ, CRY1/2, Casein Kinase family, and FBXL3.

In recent decades, an increasing number of minor molecules have been found to regulate circadian rhythms with the ability to interact with either core or non-core clock proteins. For example, small molecules such as KL001, which stabilizes the CLOCK protein, and SR9009, an agonist of REV-ERB, have shown significant effects on the circadian clock mechanism. These molecules can modulate the activity of core circadian proteins and thus influence the overall circadian rhythm [298, 299] (Fig. 6). This discovery brings about a broader spectrum of treatment options for patients dealing with clock-related disorders [300]. Recombinant cortistatin (CORT) protein stabilizers have been proven to enhance glucose tolerance and extend circadian rhythm [301, 302], and conversely, inhibitors of its transcriptional repressive activity have shown a hindrance in the growth of breast cancer cell lines [303]. Emerging studies report that the pharmacological suppression of RORγ exhibits considerable anti-tumor efficacy in various cancer types, including pancreatic and triple-negative breast cancer, both *in vitro* and *in vivo* [304, 305]. Additionally, the use of synthetic RORγ agonists has demonstrated a capacity to bolster antitumor immunity by augmenting cytotoxic lymphocyte function while decreasing immunosuppressive mechanisms [306]. XY018 and SR2211, which are RORγ-selective antagonists, were found to inhibit the proliferation of prostate cancer cells expressing the androgen receptor. Furthermore, these antagonists restored sensitivity to treatment with enzalutamide, a popular androgen inhibitor for prostate cancer. Enzalutamide induces apoptosis and reduces the expression of key proliferation and survival proteins, both of which have been identified as critical events in treating this condition [304]. RORγ agonists have been demonstrated to be effective in modulating diverse signaling pathways to improve the immune system's antitumor response to leukemia, colon cancer, and breast cancer [307-309]. LYC-55716, a ROR receptor agonist, has undergone rigorous preclinical and clinical testing and has shown excellent tolerability, safety, and pharmacokinetics in isolation or when combined with conventional immunotherapy drugs [310]. Nobiletin has been identified as an agonist of RORα and RORγ, exhibiting various crucial anticancer properties. This compound exhibits the potential to initiate apoptosis and cell cycle arrest, while also upregulating tumor suppressors and inhibiting the expression of oncogenic factors. Furthermore, it can suppress migration and invasion while increasing chemosensitivity in various types of cancer cells [311]. These findings substantiate the hypothesis that ROR subtypes could be a valuable target for developing effective cancer treatments.

The function of CRY1 and CRY2 in suppressing clock-regulated gene transcription through direct interaction with the BMAL1/CLOCK complex is a significant aspect worth noting [312-315]. KL001 was identified as the first compound to bind to and stabilize CRY by preventing FBXL3-mediated ubiquitin-dependent degradation [301]. According to recent research, administration of KL001 has been shown to decrease the expression of OLIG2 and SOX2 in healthy brain cells, deter the proliferation of GSCs, and mitigate their toxicity [316]. Intriguingly, in vitro assessments indicated that pharmacologically targeting CRE through KS15 resulted in diminished proliferation among MCF-7 breast cancer cells, as well as heightened receptivity to doxorubicin and tamoxifen. Conversely, no harmful effects were observed in normal breast epithelial MCF-10A cells [303]. Given these contradictory outcomes, it is imperative that further research is conducted to discern whether CRY holds potential as a new therapeutic avenue.

The nuclear receptors, REV-ERBα and REV-ERBβ, play a pivotal role not only in the molecular circadian clock but also in metabolic regulation [9, 317]. The expression of REV-ERBS irregular in various cancer types, where it primarily influences blood glucose levels, blood lipids, and energy metabolism [318]. Notably, the administration of REV-ERB agonists elicits a dramatic response in mice, including the selective destruction of cancer cells [319]. The antitumor effects of REV-ERB agonists SR9009 and SR9011 were further confirmed by subsequent studies, and treatment with these drugs resulted in a significant reduction in the proliferation and viability of glioma cells, while also inhibiting the growth of both chemoresistant and chemosensitive tumors by impeding autophagy in lung cancer cells [320, 321]. Additional studies have demonstrated that autophagy and de novo lipogenesis play critical roles in eliciting apoptotic responses in malignant cells treated with SR9009 and SR9011 [319]. Additional chemical agonists directed towards REV-ERBα, such as GSK2945, GSK0999, GSK5072, and GSK2667, have undergone development. However, further investigation is necessary to determine their impact on cancer [322].

Casein kinases 1δ and 1ε (CK1δ/ε) are key components of the circadian clock, regulating their timed nuclear import activity through the phosphorylation of PERs to determine circadian rhythms [323]. In recent years, casein kinases have been recognized as oncogenic proteins and have become therapeutic targets for cancer [324]. A series of potent and selective CK1δ/ε inhibitors have been demonstrated to have antitumor effects on breast cancer both *in vivo* and *in vitro* [325]. Additionally, a separate investigation revealed that the CK1δ/ε inhibitor IC261 exhibited significant suppression of colon, liver, and other human cancer cell survival and proliferation while promoting apoptosis, a key factor in multiple carcinogenic mechanisms [326]. Apart from research initiatives aimed towards the CK1 family, a recent study utilizing cell-based screening techniques has highlighted the discovery of a novel and efficacious CK2 inhibitor (GO289), exhibiting exceptional potency and selectivity. Its application has been found to considerably extend circadian rhythms and impede the proliferation of various human and mouse cancer cells [327]. A number of preclinical and clinical studies have indicated that several anticancer drugs targeted at the CK1 or CK2 family, such as umbralisib and CX4945, exhibit distinct immunomodulatory effects in the management of hematological malignancies such as chronic lymphocytic leukemia, myeloid dysplastic syndromes, acute myeloid leukemia and multiple myeloma (MM) [328, 329]. Altogether, these results suggest that targeting CK family members is a promising therapeutic approach for the treatment of various cancers.

### 6.3 Circadian rhythm and hormone

Several experimental and clinical studies have demonstrated that melatonin possesses strong anticancer properties in conjunction with non-pharmaceutical interventions [330]. A meta-analysis showed that low melatonin secretion was associated with higher cancer incidence in patients exposed to light at night [331]. Furthermore, it should be noted that melatonin demonstrates a targeted affinity towards cancer cells, while leaving normal cells unaffected [332]. Additionally, melatonin serves to mitigate the harmful impact of chemotherapy on normal cells by restoring the light/dark circadian rhythm [333]. These findings suggest that melatonin therapy has the potential to become a highly secure chronobiological approach to treating cancer. Similar to melatonin, glucocorticoid (GC) is also an anticancer hormone [334]. GCs show remarkable efficacy in treating lymphoid malignancies such as leukemia, lymphoma, and multiple myeloma. Significant efforts have been made to augment their effects and surmount drug resistance [335]. Intriguingly, recent research highlights that enhanced circadian clock treatment with dexamethasone in mice reduces melanoma tumors [336]. Although GCs have a tumor-suppressive role, there have been recent clinical observations indicating that they may not be responsive to immune checkpoint inhibitors (such as anti-PD-L1), possibly due to their immunosuppressive effect resulting in inadequate tumor proliferation and growth [310]. Given the diurnal fluctuations in endogenous GC levels, it is imperative to take into account the dose and administration timing of corticosteroids to enhance the effectiveness and safety of immune-based cancer therapy.

## Conclusion

Over the past few decades, extensive chronobiological research has significantly increased our comprehension of the operative functions and mechanisms of circadian clocks in human health and disease, specifically cancer. Interference with circadian rhythms can have adverse effects on both the molecular clocks of tumors and the host's circadian systems, thereby increasing the risk and progression of cancer. It is plausible to suggest that multiple pathways controlled by the circadian clock are utilized by cancer cells as they consume nutrients at a high metabolic rate. This article examines the interdependent relationship between non-clock transcription factors and circadian rhythm-related hormones with the circadian clock in order to establish the correlation between circadian rhythm and tumor metabolism. The circadian rhythm exerts a significant influence on the immune system and its response to tumor cells. Moreover, it is plausible that CRD may facilitate tumor growth and immune evasion by regulating cell cycle regulators and immune response. Further, we provide an overview of the potential application of circadian rhythm in tumor therapy, such as chronochemotherapy and targeting biological clock components and related hormones. However, current research is in its nascent stage, and more clinical trials are necessary to validate its benefits. Given that endocrine tissues play a pivotal role in the circadian regulation of systemic physiology, diagnosing and treating circadian endocrine imbalances could enhance the wellbeing of cancer survivors. In summary, the integration of circadian rhythms with medicine holds enormous potential to enhance cancer care and prevention by optimizing treatment timing, improving therapeutic efficacy, and reducing side effects, ultimately leading to better patient outcomes and quality of life.

## References

[1] Fu L, Lee CC (2003). The circadian clock: pacemaker and tumour suppressor. Nature reviews. Cancer, 3:350-61. 10.1038/nrc1072 12724733

[2] Sahar S, Sassone-Corsi P (2009). Metabolism and cancer: the circadian clock connection. Nature reviews. Cancer, 9:886-96. 10.1038/nrc2747 19935677

[3] Masri S, Kinouchi K, Sassone-Corsi P (2015). Circadian clocks, epigenetics, and cancer. Curr Opin Oncol, 27:50-6. 10.1097/CCO.0000000000000153 PMC4732884 25405464

[4] Lie JS, Roessink J, Kjaerheim K (2006). Breast cancer and night work among norwegian nurses. CCC, 17:39-44. 10.1007/s10552-005-3639-2 16411051

[5] Papantoniou K, Castaño-Vinyals G, Espinosa A, Aragonés N, Pérez-Gómez B, Burgos J, et al (2015). Night shift work, chronotype and prostate cancer risk in the mcc-spain case-control study. Int J Cancer, 137:1147-57. 10.1002/ijc.29400 25530021

[6] Schernhammer ES, Laden F, Speizer FE, Willett WC, Hunter DJ, Kawachi I, et al (2001). Rotating night shifts and risk of breast cancer in women participating in the nurses' health study. Journal of the National Cancer Institute, 93:1563-8. 10.1093/jnci/93.20.1563 11604480

[7] Masri S, Sassone-Corsi P (2018). The emerging link between cancer, metabolism, and circadian rhythms. Nat Med, 24:1795-803. 10.1038/s41591-018-0271-8 PMC6535395 30523327

[8] Kinouchi K, Sassone-Corsi P (2020). Metabolic rivalry: circadian homeostasis and tumorigenesis. Nature reviews. Cancer, 20:645-61. 10.1038/s41568-020-0291-9 32895495

[9] Solt LA, Wang Y, Banerjee S, Hughes T, Kojetin DJ, Lundasen T, et al (2012). Regulation of circadian behaviour and metabolism by synthetic rev-erb agonists. Nature, 485:62-8. 10.1038/nature11030 PMC3343186 22460951

[10] Malik S, Stokes J III, Manne U, Singh R, Mishra MK (2022). Understanding the significance of biological clock and its impact on cancer incidence. Cancer Lett, 527:80-94. 10.1016/j.canlet.2021.12.006 PMC8816870 34906624

[11] Lévi F, Okyar A, Dulong S, Innominato PF, Clairambault J (2010). Circadian timing in cancer treatments. Annu Rev Pharmacol Toxicol, 50:377-421. 10.1146/annurev.pharmtox.48.113006.094626 20055686

[12] Ruan W, Yuan X, Eltzschig HK (2021). Circadian rhythm as a therapeutic target. Nature reviews. Drug discovery, 20:287-307. 10.1038/s41573-020-00109-w PMC8525418 33589815

[13] Lee Y (2021). Roles of circadian clocks in cancer pathogenesis and treatment. Experimental & molecular medicine, 53:1529-38. 10.1038/s12276-021-00681-0 PMC8568965 34615982

[14] Wang C, Barnoud C, Cenerenti M, Sun M, Caffa I, Kizil B, et al (2023). Dendritic cells direct circadian anti-tumour immune responses. Nature, 614:136-43. 10.1038/s41586-022-05605-0 PMC9891997 36470303

[15] Aiello I, Fedele MLM, Román F, Marpegan L, Caldart C, Chiesa JJ, et al (2020). Circadian disruption promotes tumor-immune microenvironment remodeling favoring tumor cell proliferation. Sci Adv, 6. 10.1126/sciadv.aaz4530 PMC7556830 33055171

[16] Petković M, Henis M, Heese O, Relógio A (2023). Chronotherapy in glioblastoma: state of the art and future perspectives. EBioMedicine, 89:104470. 10.1016/j.ebiom.2023.104470 PMC9958380 36796229

[17] Lévi FA, Okyar A, Hadadi E, Innominato PF, Ballesta A (2024). Circadian regulation of drug responses: toward sex-specific and personalized chronotherapy. Annu Rev Pharmacol Toxicol, 64:89-114. 10.1146/annurev-pharmtox-051920-095416 37722720

[18] Chan P, Rich JN, Kay SA (2023). Watching the clock in glioblastoma. Neuro Oncol, 25:1932-46. 10.1093/neuonc/noad107 PMC10628946 37326042

[19] Kisamore CO, Elliott BD, DeVries AC, Nelson RJ, Walker WHN (2023). Chronotherapeutics for solid tumors. Pharmaceutics, 15. 10.3390/pharmaceutics15082023 PMC10459260 37631237

[20] Zhu X, Maier G, Panda S (2024). Learning from circadian rhythm to transform cancer prevention, prognosis, and survivorship care. Trends Cancer, 10:196-207. 10.1016/j.trecan.2023.11.002 PMC10939944 38001006

[21] Honma S (2018). The mammalian circadian system: a hierarchical multi-oscillator structure for generating circadian rhythm. The journal of physiological sciences: JPS, 68:207-19. 10.1007/s12576-018-0597-5 PMC10717972 29460036

[22] Fang B, Everett LJ, Jager J, Briggs E, Armour SM, Feng D, et al (2014). Circadian enhancers coordinate multiple phases of rhythmic gene transcription in vivo. Cell, 159:1140-52. 10.1016/j.cell.2014.10.022 PMC4243056 25416951

[23] Panda S, Antoch MP, Miller BH, Su AI, Schook AB, Straume M, et al (2002). Coordinated transcription of key pathways in the mouse by the circadian clock., 307-20. 10.1016/s0092-8674(02)00722-5 12015981

[24] Gentry NW, Ashbrook LH, Fu Y, Ptáček LJ (2021). Human circadian variations. The Journal of clinical investigation, 131. 10.1172/JCI148282 PMC8363277 34396981

[25] Cox KH, Takahashi JS (2019). Circadian clock genes and the transcriptional architecture of the clock mechanism. J Mol Endocrinol, 63:R93-102. 10.1530/JME-19-0153 PMC6872945 31557726

[26] Zimmet P, Alberti KGMM, Stern N, Bilu C, El-Osta A, Einat H, et al (2019). The circadian syndrome: is the metabolic syndrome and much more!. J Intern Med, 286:181-91. 10.1111/joim.12924 PMC6851668 31081577

[27] Sheikh-Ali M, Maharaj J (2014). Circadian clock desynchronisation and metabolic syndrome. Postgrad Med J, 90:461-6. 10.1136/postgradmedj-2013-132366 24958893

[28] Eckel-Mahan K, Sassone-Corsi P (2013). Metabolism and the circadian clock converge. Physiol Rev, 93:107-35. 10.1152/physrev.00016.2012 PMC3781773 23303907

[29] Fatima N, Rana S (2020). Metabolic implications of circadian disruption. Pflugers Archiv : European journal of physiology, 472:513-26. 10.1007/s00424-020-02381-6 32363530

[30] Cagampang FR, Bruce KD (2012). The role of the circadian clock system in nutrition and metabolism. The British journal of nutrition, 108:381-92. 10.1017/S0007114512002139 22676899

[31] Mazzoccoli G, Pazienza V, Vinciguerra M (2012). Clock genes and clock-controlled genes in the regulation of metabolic rhythms. Chronobiol Int, 29:227-51. 10.3109/07420528.2012.658127 22390237

[32] Delezie J, Challet E (2011). Interactions between metabolism and circadian clocks: reciprocal disturbances. Ann N Y Acad Sci, 1243:30-46. 10.1111/j.1749-6632.2011.06246.x 22211891

[33] Saez MC, Barriga C, Garcia JJ, Rodríguez AB, Ortega E (2005). Effect of the preventive-therapeutic administration of melatonin on mammary tumour-bearing animals. Mol Cell Biochem, 268:25-31. 10.1007/s11010-005-2994-3 15724434

[34] Filipski E, King VM, Li X, Granda TG, Mormont M, Liu X, et al (2002). Host circadian clock as a control point in tumor progression. Journal of the National Cancer Institute, 94:690-7. 10.1093/jnci/94.9.690 11983758

[35] Filipski E, Lévi F (2009). Circadian disruption in experimental cancer processes. Integr Cancer Ther, 8:298-302. 10.1177/1534735409352085 20042408

[36] Patke A, Young MW, Axelrod S (2020). Molecular mechanisms and physiological importance of circadian rhythms. Nature reviews. Molecular cell biology, 21:67-84. 10.1038/s41580-019-0179-2 31768006

[37] Konopka RJ, Benzer S (1971). Clock mutants of drosophila melanogaster. Proc Natl Acad Sci U S A, 68:2112-6. 10.1073/pnas.68.9.2112 PMC389363 5002428

[38] Hardin PE, Hall JC, Rosbash M (1990). Feedback of the drosophila period gene product on circadian cycling of its messenger rna levels. Nature, 343:536-40. 10.1038/343536a0 2105471

[39] King DP, Zhao Y, Sangoram AM, Wilsbacher LD, Tanaka M, Antoch MP, et al (1997). Positional cloning of the mouse circadian clock gene. Cell, 89:641-53. 10.1016/s0092-8674(00)80245-7 PMC3815553 9160755

[40] Logan RW, McClung CA (2019). Rhythms of life: circadian disruption and brain disorders across the lifespan. Nature reviews. Neuroscience, 20:49-65. 10.1038/s41583-018-0088-y PMC6338075 30459365

[41] Allada R, Bass J (2021). Circadian mechanisms in medicine. The New England journal of medicine, 384:550-61. 10.1056/NEJMra1802337 PMC8108270 33567194

[42] Musiek ES, Holtzman DM (2016). Mechanisms linking circadian clocks, sleep, and neurodegeneration. Science (New York, N.Y.), 354:1004-8. 10.1126/science.aah4968 PMC5219881 27885006

[43] Preitner N, Damiola F, Lopez-Molina L, Zakany J, Duboule D, Albrecht U, et al (2002). The orphan nuclear receptor rev-erbalpha controls circadian transcription within the positive limb of the mammalian circadian oscillator. Cell, 110:251-60. 10.1016/s0092-8674(02)00825-5 12150932

[44] Zhang Z, Zeng P, Gao W, Zhou Q, Feng T, Tian X (2021). Circadian clock: a regulator of the immunity in cancer. Cell communication and signaling : CCS, 19:37. 10.1186/s12964-021-00721-2 PMC7986390 33752691

[45] Relógio A, Thomas P, Medina-Pérez P, Reischl S, Bervoets S, Gloc E, et al (2014). Ras-mediated deregulation of the circadian clock in cancer. PLoS Genet, 10:e1004338. 10.1371/journal.pgen.1004338 PMC4038477 24875049

[46] Shafi AA, Knudsen KE (2019). Cancer and the circadian clock. Cancer Res, 79:3806-14. 10.1158/0008-5472.CAN-19-0566 PMC8121183 31300477

[47] Soták M, Sumová A, Pácha J (2014). Cross-talk between the circadian clock and the cell cycle in cancer. Ann Med, 46:221-32. 10.3109/07853890.2014.892296 24779962

[48] Kettner NM, Katchy CA, Fu L (2014). Circadian gene variants in cancer. Ann Med, 46:208-20. 10.3109/07853890.2014.914808 PMC4153443 24901356

[49] Fu L, Kettner NM (2013). The circadian clock in cancer development and therapy. Prog Mol Biol Transl Sci, 119:221-82. 10.1016/B978-0-12-396971-2.00009-9 PMC4103166 23899600

[50] Verlande A, Masri S (2019). Circadian clocks and cancer: timekeeping governs cellular metabolism. Trends in endocrinology and metabolism: TEM, 30:445-58. 10.1016/j.tem.2019.05.001 PMC6679985 31155396

[51] Klevecz RR, Shymko RM, Blumenfeld D, Braly PS (1987). Circadian gating of s phase in human ovarian cancer. Cancer Res, 47:6267-71. 3677075

[52] You S, Wood PA, Xiong Y, Kobayashi M, Du-Quiton J, Hrushesky WJM (2005). Daily coordination of cancer growth and circadian clock gene expression. Breast Cancer Res Treat, 91:47-60. 10.1007/s10549-004-6603-z 15868431

[53] Brandi G, Calabrese C, Pantaleo MA, Morselli Labate A, Di Febo G, Hakim R, et al (2004). Circadian variations of rectal cell proliferation in patients affected by advanced colorectal cancer. Cancer Lett, 208:193-6. 10.1016/j.canlet.2003.11.015 15142678

[54] Sedivy R, Thurner S, Budinsky AC, Köstler WJ, Zielinski CC (2002). Short-term rhythmic proliferation of human breast cancer cell lines: surface effects and fractal growth patterns. The Journal of pathology, 197:163-9. 10.1002/path.1118 12015739

[55] Xie Y, Tang Q, Chen G, Xie M, Yu S, Zhao J, et al (2019). New insights into the circadian rhythm and its related diseases. Front Physiol, 10:682. 10.3389/fphys.2019.00682 PMC6603140 31293431

[56] Neves AR, Albuquerque T, Quintela T, Costa D (2022). Circadian rhythm and disease: relationship, new insights, and future perspectives. J Cell Physiol, 237:3239-56. 10.1002/jcp.30815 35696609

[57] Schernhammer ES, Laden F, Speizer FE, Willett WC, Hunter DJ, Kawachi I, et al (2003). Night-shift work and risk of colorectal cancer in the nurses' health study. Journal of the National Cancer Institute, 95:825-8. 10.1093/jnci/95.11.825 12783938

[58] Kubo T, Ozasa K, Mikami K, Wakai K, Fujino Y, Watanabe Y, et al (2006). Prospective cohort study of the risk of prostate cancer among rotating-shift workers: findings from the japan collaborative cohort study. Am J Epidemiol, 164:549-55. 10.1093/aje/kwj232 16829554

[59] Lahti TA, Partonen T, Kyyrönen P, Kauppinen T, Pukkala E (2008). Night-time work predisposes to non-hodgkin lymphoma. Int J Cancer, 123:2148-51. 10.1002/ijc.23566 18697199

[60] Hansen J, Lassen CF (2012). Nested case-control study of night shift work and breast cancer risk among women in the danish military. Occup Environ Med, 69:551-6. 10.1136/oemed-2011-100240 22645325

[61] Hansen J, Stevens RG (2012). Case-control study of shift-work and breast cancer risk in danish nurses: impact of shift systems. European journal of cancer (Oxford, England : 1990), 48:1722-9. 10.1016/j.ejca.2011.07.005 21852111

[62] Lie JS, Kjuus H, Zienolddiny S, Haugen A, Stevens RG, Kjærheim K (2011). Night work and breast cancer risk among norwegian nurses: assessment by different exposure metrics. Am J Epidemiol, 173:1272-9. 10.1093/aje/kwr014 21454824

[63] Stevens RG (2009). Working against our endogenous circadian clock: breast cancer and electric lighting in the modern world. Mutation research, 680:106-8. 10.1016/j.mrgentox.2009.08.004 20336819

[64] Touitou Y, Bogdan A, Lévi F, Benavides M, Auzéby A (1996). Disruption of the circadian patterns of serum cortisol in breast and ovarian cancer patients: relationships with tumour marker antigens. Br J Cancer, 74:1248-52. 10.1038/bjc.1996.524 PMC2075940 8883412

[65] Panzer A (1997). Melatonin in osteosarcoma: an effective drug? Med Hypotheses, 48:523-5. 10.1016/s0306-9877(97)90123-7 9247897

[66] Bhatti P, Mirick DK, Davis S (2012). Invited commentary: shift work and cancer., 760-3, 764-5. 10.1093/aje/kws311 23035018

[67] Haus EL, Smolensky MH (2013). Shift work and cancer risk: potential mechanistic roles of circadian disruption, light at night, and sleep deprivation. Sleep Med Rev, 17:273-84. 10.1016/j.smrv.2012.08.003 23137527

[68] Buzzelli G, Dattolo P, Pinzani M, Brocchi A, Romano S, Gentilini P (1993). Circulating growth hormone and insulin-like growth factor-i in nonalcoholic liver cirrhosis with or without superimposed hepatocarcinoma: evidence of an altered circadian rhythm. The American journal of gastroenterology, 88:1744-8. 8213718

[69] Davis S, Mirick DK, Stevens RG (2001). Night shift work, light at night, and risk of breast cancer., 1557-62. 10.1093/jnci/93.20.1557 11604479

[70] Aihara T, Miyoshi Y, Koyama K, Suzuki M, Takahashi E, Monden M, et al (1998). Cloning and mapping of smarca5 encoding hsnf2h, a novel human homologue of drosophila iswi. Cytogenetics and cell genetics, 81:191-3. 10.1159/000015027 9730600

[71] Narasimamurthy R, Hatori M, Nayak SK, Liu F, Panda S, Verma IM (2012). Circadian clock protein cryptochrome regulates the expression of proinflammatory cytokines. Proc Natl Acad Sci U S A, 109:12662-7. 10.1073/pnas.1209965109 PMC3411996 22778400

[72] Blask DE, Hill SM, Dauchy RT, Xiang S, Yuan L, Duplessis T, et al (2011). Circadian regulation of molecular, dietary, and metabolic signaling mechanisms of human breast cancer growth by the nocturnal melatonin signal and the consequences of its disruption by light at night. J Pineal Res, 51:259-69. 10.1111/j.1600-079X.2011.00888.x PMC3162043 21605163

[73] Kuo S, Chen S, Yeh K, Hou M, Chang Y, Hsu NC, et al (2009). Disturbance of circadian gene expression in breast cancer. Virchows Archiv : an international journal of pathology, 454:467-74. 10.1007/s00428-009-0761-7 19296127

[74] Hoffman AE, Yi C, Zheng T, Stevens RG, Leaderer D, Zhang Y, et al (2010). Clock in breast tumorigenesis: genetic, epigenetic, and transcriptional profiling analyses. Cancer Res, 70:1459-68. 10.1158/0008-5472.CAN-09-3798 PMC3188957 20124474

[75] Zhu Y, Stevens RG, Leaderer D, Hoffman A, Holford T, Zhang Y, et al (2008). Non-synonymous polymorphisms in the circadian gene npas2 and breast cancer risk. Breast Cancer Res Treat, 107:421-5. 10.1007/s10549-007-9565-0 PMC2366999 17453337

[76] Dai H, Zhang L, Cao M, Song F, Zheng H, Zhu X, et al (2011). The role of polymorphisms in circadian pathway genes in breast tumorigenesis. Breast Cancer Res Treat, 127:531-40. 10.1007/s10549-010-1231-2 20978934

[77] Chen S, Choo K, Hou M, Yeh K, Kuo S, Chang J (2005). Deregulated expression of the per1, per2 and per3 genes in breast cancers. Carcinogenesis, 26:1241-6. 10.1093/carcin/bgi075 15790588

[78] Winter SL, Bosnoyan-Collins L, Pinnaduwage D, Andrulis IL (2007). Expression of the circadian clock genes per1 and per2 in sporadic and familial breast tumors. Neoplasia (New York, N.Y.), 9:797-800. 10.1593/neo.07595 PMC2040206 17971899

[79] Hua H, Wang Y, Wan C, Liu Y, Zhu B, Yang C, et al (2006). Circadian gene mper2 overexpression induces cancer cell apoptosis. Cancer Sci, 97:589-96. 10.1111/j.1349-7006.2006.00225.x PMC2662332 16827798

[80] Xiang S, Coffelt SB, Mao L, Yuan L, Cheng Q, Hill SM (2008). Period-2: a tumor suppressor gene in breast cancer. Journal of circadian rhythms, 6:4. 10.1186/1740-3391-6-4 PMC2365929 18334030

[81] Antoch MP, Gorbacheva VY, Vykhovanets O, Toshkov IA, Kondratov RV, Kondratova AA, et al (2008). Disruption of the circadian clock due to the clock mutation has discrete effects on aging and carcinogenesis. Cell cycle (Georgetown, Tex.), 7:1197-204. 10.4161/cc.7.9.5886 PMC2744375 18418054

[82] Nakagawa H, Koyanagi S, Kuramoto Y, Yoshizumi A, Matsunaga N, Shimeno H, et al (2008). Modulation of circadian rhythm of dna synthesis in tumor cells by inhibiting platelet-derived growth factor signaling. J Pharmacol Sci, 107:401-7. 10.1254/jphs.08080fp 18678981

[83] van Zijl F, Mair M, Csiszar A, Schneller D, Zulehner G, Huber H, et al (2009). Hepatic tumor-stroma crosstalk guides epithelial to mesenchymal transition at the tumor edge. Oncogene, 28:4022-33. 10.1038/onc.2009.253 PMC2900602 19718050

[84] Jung-Hynes B, Schmit TL, Reagan-Shaw SR, Siddiqui IA, Mukhtar H, Ahmad N (2011). Melatonin, a novel sirt1 inhibitor, imparts antiproliferative effects against prostate cancer in vitro in culture and in vivo in tramp model. J Pineal Res, 50:140-9. 10.1111/j.1600-079X.2010.00823.x PMC3052633 21062352

[85] Kloog I, Haim A, Stevens RG, Portnov BA (2009). Global co-distribution of light at night (lan) and cancers of prostate, colon, and lung in men. Chronobiol Int, 26:108-25. 10.1080/07420520802694020 19142761

[86] Eisele L, Prinz R, Klein-Hitpass L, Nückel H, Lowinski K, Thomale J, et al (2009). Combined per2 and cry1 expression predicts outcome in chronic lymphocytic leukemia. Eur J Haematol, 83:320-7. 10.1111/j.1600-0609.2009.01287.x 19500131

[87] Kim KS, Kim YC, Oh IJ, Kim SS, Choi JY, Ahn RS (2012). Association of worse prognosis with an aberrant diurnal cortisol rhythm in patients with advanced lung cancer. Chronobiol Int, 29:1109-20. 10.3109/07420528.2012.706767 22889441

[88] Sephton SE, Lush E, Dedert EA, Floyd AR, Rebholz WN, Dhabhar FS, et al (2013). Diurnal cortisol rhythm as a predictor of lung cancer survival. Brain, behavior, and immunity, 30 Suppl:S163-70. 10.1016/j.bbi.2012.07.019 22884416

[89] Zhou F, He X, Liu H, Zhu Y, Jin T, Chen C, et al (2012). Functional polymorphisms of circadian positive feedback regulation genes and clinical outcome of chinese patients with resected colorectal cancer. Cancer, 118:937-46. 10.1002/cncr.26348 21773969

[90] Kloog I, Haim A, Stevens RG, Portnov BA (2009). Global co-distribution of light at night (lan) and cancers of prostate, colon, and lung in men. Chronobiol Int, 26:108-25. 10.1080/07420520802694020 19142761

[91] Gery S, Komatsu N, Baldjyan L, Yu A, Koo D, Koeffler HP (2006). The circadian gene per1 plays an important role in cell growth and dna damage control in human cancer cells. Mol Cell, 22:375-82. 10.1016/j.molcel.2006.03.038 16678109

[92] Gery S, Koeffler HP (2007). The role of circadian regulation in cancer. Cold Spring Harbor symposia on quantitative biology, 72:459-64. 10.1101/sqb.2007.72.004 18419305

[93] Mullenders J, Fabius AWM, Madiredjo M, Bernards R, Beijersbergen RL (2009). A large scale shrna barcode screen identifies the circadian clock component arntl as putative regulator of the p53 tumor suppressor pathway. PLoS One, 4:e4798. 10.1371/journal.pone.0004798 PMC2653142 19277210

[94] Cao Q, Gery S, Dashti A, Yin D, Zhou Y, Gu J, et al (2009). A role for the clock gene per1 in prostate cancer. Cancer Res, 69:7619-25. 10.1158/0008-5472.CAN-08-4199 PMC2756309 19752089

[95] Jung-Hynes B, Huang W, Reiter RJ, Ahmad N (2010). Melatonin resynchronizes dysregulated circadian rhythm circuitry in human prostate cancer cells. J Pineal Res, 49:60-8. 10.1111/j.1600-079X.2010.00767.x PMC3158680 20524973

[96] Miyamoto N, Izumi H, Noguchi T, Nakajima Y, Ohmiya Y, Shiota M, et al (2008). Tip60 is regulated by circadian transcription factor clock and is involved in cisplatin resistance. The Journal of biological chemistry, 283:18218-26. 10.1074/jbc.M802332200 18458078

[97] Zhu Y, Stevens RG, Hoffman AE, Fitzgerald LM, Kwon EM, Ostrander EA, et al (2009). Testing the circadian gene hypothesis in prostate cancer: a population-based case-control study. Cancer Res, 69:9315-22. 10.1158/0008-5472.CAN-09-0648 PMC2955869 19934327

[98] Wood PA, Yang X, Hrushesky WJM (2009). Clock genes and cancer. Integr Cancer Ther, 8:303-8. 10.1177/1534735409355292 20042409

[99] Xue X, Liu F, Han Y, Li P, Yuan B, Wang X, et al (2014). Silencing npas2 promotes cell growth and invasion in dld-1 cells and correlated with poor prognosis of colorectal cancer. Biochem Biophys Res Commun, 450:1058-62. 10.1016/j.bbrc.2014.06.104 24978311

[100] Innominato PF, Giacchetti S, Bjarnason GA, Focan C, Garufi C, Coudert B, et al (2012). Prediction of overall survival through circadian rest-activity monitoring during chemotherapy for metastatic colorectal cancer., 2684-92. 10.1002/ijc.27574 22488038

[101] Faustino RS, Cheung P, Richard MN, Dibrov E, Kneesch AL, Deniset JF, et al (2008). Ceramide regulation of nuclear protein import. J Lipid Res, 49:654-62. 10.1194/jlr.M700464-JLR200 18083977

[102] Viswanathan AN, Schernhammer ES (2009). Circulating melatonin and the risk of breast and endometrial cancer in women. Cancer Lett, 281:1-7. 10.1016/j.canlet.2008.11.002 PMC2735793 19070424

[103] Viswanathan AN, Hankinson SE, Schernhammer ES (2007). Night shift work and the risk of endometrial cancer. Cancer Res, 67:10618-22. 10.1158/0008-5472.CAN-07-2485 17975006

[104] Tokunaga H, Takebayashi Y, Utsunomiya H, Akahira J, Higashimoto M, Mashiko M, et al (2008). Clinicopathological significance of circadian rhythm-related gene expression levels in patients with epithelial ovarian cancer. Acta Obstet Gynecol Scand, 87:1060-70. 10.1080/00016340802348286 18720043

[105] Hsu C, Lin S, Lu C, Lin P, Yang M (2012). Altered expression of circadian clock genes in head and neck squamous cell carcinoma. Tumour biology : the journal of the International Society for Oncodevelopmental Biology and Medicine, 33:149-55. 10.1007/s13277-011-0258-2 22081375

[106] Sun C, Huang S, Zeng J, Liu D, Xiao Q, Tian W, et al (2010). Per2 inhibits k562 leukemia cell growth in vitro and in vivo through cell cycle arrest and apoptosis induction. Pathology oncology research : POR, 16:403-11. 10.1007/s12253-009-9227-0 19957060

[107] Yang WS, Stockwell BR (2008). Inhibition of casein kinase 1-epsilon induces cancer-cell-selective, period2-dependent growth arrest. Genome Biol, 9:R92. 10.1186/gb-2008-9-6-r92 PMC2481424 18518968

[108] Shih M, Yeh K, Tang K, Chen J, Chang J (2006). Promoter methylation in circadian genes of endometrial cancers detected by methylation-specific pcr. Mol Carcinog, 45:732-40. 10.1002/mc.20198 16683245

[109] Suzuki T, Sato F, Kondo J, Liu Y, Kusumi T, Fujimoto K, et al (2008). Period is involved in the proliferation of human pancreatic mia-paca2 cancer cells by tnf-alpha. Biomedical research (Tokyo, Japan), 29:99-103. 10.2220/biomedres.29.99 18480551

[110] Taniguchi H, Fernández AF, Setién F, Ropero S, Ballestar E, Villanueva A, et al (2009). Epigenetic inactivation of the circadian clock gene bmal1 in hematologic malignancies. Cancer Res, 69:8447-54. 10.1158/0008-5472.CAN-09-0551 19861541

[111] Zhu Y, Leaderer D, Guss C, Brown HN, Zhang Y, Boyle P, et al (2007). Ala394thr polymorphism in the clock gene npas2: a circadian modifier for the risk of non-hodgkin's lymphoma. Int J Cancer, 120:432-5. 10.1002/ijc.22321 PMC2375536 17096334

[112] Pukkala E, Aspholm R, Auvinen A, Eliasch H, Gundestrup M, Haldorsen T, et al (2003). Cancer incidence among 10,211 airline pilots: a nordic study. Aviation, space, and environmental medicine, 74:699-706. 12862322

[113] Relles D, Sendecki J, Chipitsyna G, Hyslop T, Yeo CJ, Arafat HA (2013). Circadian gene expression and clinicopathologic correlates in pancreatic cancer. Journal of gastrointestinal surgery : official journal of the Society for Surgery of the Alimentary Tract, 17:443-50. 10.1007/s11605-012-2112-2 23254314

[114] Lin Y, Chang JH, Yeh K, Yang M, Liu T, Lin S, et al (2008). Disturbance of circadian gene expression in hepatocellular carcinoma. Mol Carcinog, 47:925-33. 10.1002/mc.20446 18444243

[115] Luo Y, Wang F, Chen L, Chen X, Chen Z, Liu P, et al (2012). Deregulated expression of cry1 and cry2 in human gliomas. Asian Pacific journal of cancer prevention : APJCP, 13:5725-8. 10.7314/apjcp.2012.13.11.5725 23317246

[116] Yang M, Chang J, Lin P, Tang K, Chen Y, Lin HY, et al (2006). Downregulation of circadian clock genes in chronic myeloid leukemia: alternative methylation pattern of hper3. Cancer Sci, 97:1298-307. 10.1111/j.1349-7006.2006.00331.x PMC11160076 16999817

[117] Honma S, Kawamoto T, Takagi Y, Fujimoto K, Sato F, Noshiro M, et al (2002). Dec1 and dec2 are regulators of the mammalian molecular clock. Nature, 419:841-4. 10.1038/nature01123 12397359

[118] Yoshitane H, Imamura K, Okubo T, Otobe Y, Kawakami S, Ito S, et al (2022). Mtor-akt signaling in cellular clock resetting triggered by osmotic stress. Antioxid Redox Signal, 37:631-46. 10.1089/ars.2021.0059 35018792

[119] Lee J, Sul HJ, Choi H, Oh DH, Shong M (2022). Loss of thyroid gland circadian per2 rhythmicity in aged mice and its potential association with thyroid cancer development. Cell Death Dis, 13:898. 10.1038/s41419-022-05342-2 PMC9596494 36284088

[120] Cazarin J, DeRollo RE, Shahidan SNAB, Burchett JB, Mwangi D, Krishnaiah S, et al (2023). Myc disrupts transcriptional and metabolic circadian oscillations in cancer and promotes enhanced biosynthesis. PLoS Genet, 19:e1010904. 10.1371/journal.pgen.1010904 PMC10491404 37639465

[121] Miller BH, McDearmon EL, Panda S, Hayes KR, Zhang J, Andrews JL, et al (2007). Circadian and clock-controlled regulation of the mouse transcriptome and cell proliferation., 3342-7. 10.1073/pnas.0611724104 PMC1802006 17360649

[122] Burchett JB, Knudsen-Clark AM, Altman BJ (2021). Myc ran up the clock: the complex interplay between myc and the molecular circadian clock in cancer. International journal of molecular sciences, 22. 10.3390/ijms22147761 PMC8305799 34299381

[123] Altman BJ, Hsieh AL, Sengupta A, Krishnanaiah SY, Stine ZE, Walton ZE, et al (2015). Myc disrupts the circadian clock and metabolism in cancer cells. Cell Metab, 22:1009-19. 10.1016/j.cmet.2015.09.003 PMC4818967 26387865

[124] Lee S, Donehower LA, Herron AJ, Moore DD, Fu L (2010). Disrupting circadian homeostasis of sympathetic signaling promotes tumor development in mice. PLoS One, 5:e10995. 10.1371/journal.pone.0010995 PMC2881876 20539819

[125] Papagiannakopoulos T, Bauer MR, Davidson SM, Heimann M, Subbaraj L, Bhutkar A, et al (2016). Circadian rhythm disruption promotes lung tumorigenesis. Cell Metab, 24:324-31. 10.1016/j.cmet.2016.07.001 PMC5367626 27476975

[126] Yang X, Wood PA, Ansell C, Hrushesky WJM (2009). Circadian time-dependent tumor suppressor function of period genes. Integr Cancer Ther, 8:309-16. 10.1177/1534735409352083 19926612

[127] Blakeman V, Williams JL, Meng Q, Streuli CH (2016). Circadian clocks and breast cancer. Breast cancer research : BCR, 18:89. 10.1186/s13058-016-0743-z PMC5010688 27590298

[128] Leonhardt G (2020). Circadian clocks in breast cancer., 3603-4. 10.1007/s00204-020-02890-4 32918561

[129] Pogue-Geile KL, Lyons-Weiler J, Whitcomb DC (2006). Molecular overlap of fly circadian rhythms and human pancreatic cancer., 55-7. 10.1016/j.canlet.2005.11.049 16451817

[130] Oda A, Katayose Y, Yabuuchi S, Yamamoto K, Mizuma M, Shirasou S, et al (2009). Clock gene mouse period2 overexpression inhibits growth of human pancreatic cancer cells and has synergistic effect with cisplatin. Anticancer Res, 29:1201-9. 19414365

[131] Fujioka A, Takashima N, Shigeyoshi Y (2006). Circadian rhythm generation in a glioma cell line. Biochem Biophys Res Commun, 346:169-74. 10.1016/j.bbrc.2006.05.094 16750513

[132] Xia H, Niu Z, Ma H, Cao S, Hao S, Liu Z, et al (2010). Deregulated expression of the per1 and per2 in human gliomas. The Canadian journal of neurological sciences. Le journal canadien des sciences neurologiques, 37:365-70. 10.1017/s031716710001026x 20481271

[133] Yang X, Wood PA, Ansell CM, Quiton DFT, Oh E, Du-Quiton J, et al (2009). The circadian clock gene per1 suppresses cancer cell proliferation and tumor growth at specific times of day. Chronobiol Int, 26:1323-39. 10.3109/07420520903431301 19916834

[134] Repouskou A, Sourlingas TG, Sekeri-Pataryas KE, Prombona A (2010). The circadian expression of c-myc is modulated by the histone deacetylase inhibitor trichostatin a in synchronized murine neuroblastoma cells. Chronobiol Int, 27:722-41. 10.3109/07420521003786800 20560708

[135] Yang X, Wood PA, Oh E, Du-Quiton J, Ansell CM, Hrushesky WJM (2009). Down regulation of circadian clock gene period 2 accelerates breast cancer growth by altering its daily growth rhythm. Breast Cancer Res Treat, 117:423-31. 10.1007/s10549-008-0133-z 18651214

[136] Zhao H, Zeng Z, Yang J, Jin Y, Qiu M, Hu X, et al (2014). Prognostic relevance of period1 (per1) and period2 (per2) expression in human gastric cancer. Int J Clin Exp Pathol, 7:619-30. PMC3925906 24551282

[137] Xuan W, Khan F, James CD, Heimberger AB, Lesniak MS, Chen P (2021). Circadian regulation of cancer cell and tumor microenvironment crosstalk. Trends Cell Biol, 31:940-50. 10.1016/j.tcb.2021.06.008 PMC8526375 34272133

[138] Yao J, He C, Zhao W, Hu N, Long D (2021). Circadian clock and cell cycle: cancer and chronotherapy. Acta Histochem, 123:151816. 10.1016/j.acthis.2021.151816 34800857

[139] Bellet MM, Stincardini C, Costantini C, Gargaro M, Pieroni S, Castelli M, et al (2021). The circadian protein per1 modulates the cellular response to anticancer treatments. International journal of molecular sciences, 22. 10.3390/ijms22062974 PMC8001324 33804124

[140] Masuda S, Narasimamurthy R, Yoshitane H, Kim JK, Fukada Y, Virshup DM (2020). Mutation of a per2 phosphodegron perturbs the circadian phosphoswitch. Proc Natl Acad Sci U S A, 117:10888-96. 10.1073/pnas.2000266117 PMC7245125 32354999

[141] Huber A, Papp SJ, Chan AB, Henriksson E, Jordan SD, Kriebs A, et al (2016). Cry2 and fbxl3 cooperatively degrade c-myc. Mol Cell, 64:774-89. 10.1016/j.molcel.2016.10.012 PMC5123859 27840026

[142] Cadenas C, van de Sandt L, Edlund K, Lohr M, Hellwig B, Marchan R, et al (2014). Loss of circadian clock gene expression is associated with tumor progression in breast cancer. Cell cycle (Georgetown, Tex.), 13:3282-91. 10.4161/15384101.2014.954454 PMC4613905 25485508

[143] Yang S, Yu C, Jiang J, Liu L, Fang X, Wu C (2014). Hepatitis b virus x protein disrupts the balance of the expression of circadian rhythm genes in hepatocellular carcinoma. Oncol Lett, 8:2715-20. 10.3892/ol.2014.2570 PMC4214404 25360177

[144] Karantanos T, Theodoropoulos G, Gazouli M, Vaiopoulou A, Karantanou C, Lymberi M, et al (2013). Expression of clock genes in patients with colorectal cancer. The International journal of biological markers, 28:280-5. 10.5301/jbm.5000033 23712462

[145] Otálora BB, Madrid JA, Alvarez N, Vicente V, Rol MA (2008). Effects of exogenous melatonin and circadian synchronization on tumor progression in melanoma-bearing c57bl6 mice. J Pineal Res, 44:307-15. 10.1111/j.1600-079X.2007.00531.x 18339126

[146] Røe OD, Anderssen E, Helge E, Pettersen CH, Olsen KS, Sandeck H, et al (2009). Genome-wide profile of pleural mesothelioma versus parietal and visceral pleura: the emerging gene portrait of the mesothelioma phenotype. PLoS One, 4:e6554. 10.1371/journal.pone.0006554 PMC2717215 19662092

[147] Arvanitis C, Felsher DW (2006). Conditional transgenic models define how myc initiates and maintains tumorigenesis. Semin Cancer Biol, 16:313-7. 10.1016/j.semcancer.2006.07.012 16935001

[148] Crusio KM, King B, Reavie LB, Aifantis I (2010). The ubiquitous nature of cancer: the role of the scf(fbw7) complex in development and transformation. Oncogene, 29:4865-73. 10.1038/onc.2010.222 PMC3651593 20543859

[149] Papp SJ, Huber A, Jordan SD, Kriebs A, Nguyen M, Moresco JJ, et al (2015). Dna damage shifts circadian clock time via hausp-dependent cry1 stabilization. Elife, 4. 10.7554/eLife.04883 PMC4352707 25756610

[150] Reitsma JM, Liu X, Reichermeier KM, Moradian A, Sweredoski MJ, Hess S, et al (2017). Composition and regulation of the cellular repertoire of scf ubiquitin ligases. Cell, 171:1326-39. 10.1016/j.cell.2017.10.016 PMC5711595 29103612

[151] Repouskou A, Prombona A (2016). C-myc targets the central oscillator gene per1 and is regulated by the circadian clock at the post-transcriptional level. Biochimica et biophysica acta, 1859:541-52. 10.1016/j.bbagrm.2016.02.001 26850841

[152] Shostak A, Ruppert B, Ha N, Bruns P, Toprak UH, Eils R, et al (2016). Myc/miz1-dependent gene repression inversely coordinates the circadian clock with cell cycle and proliferation. Nat Commun, 7:11807. 10.1038/ncomms11807 PMC4931031 27339797

[153] Fu L, Pelicano H, Liu J, Huang P, Lee C (2002). The circadian gene period2 plays an important role in tumor suppression and dna damage response in vivo. Cell, 111:41-50. 10.1016/s0092-8674(02)00961-3 12372299

[154] Grabocka E, Pylayeva-Gupta Y, Jones MJK, Lubkov V, Yemanaberhan E, Taylor L, et al (2014). Wild-type h- and n-ras promote mutant k-ras-driven tumorigenesis by modulating the dna damage response. Cancer Cell, 25:243-56. 10.1016/j.ccr.2014.01.005 PMC4063560 24525237

[155] Lv J, Wang J, Chang S, Liu M, Pang X (2016). The greedy nature of mutant ras: a boon for drug discovery targeting cancer metabolism? Acta Biochim Biophys Sin (Shanghai), 48:17-26. 10.1093/abbs/gmv102 26487443

[156] Kimmelman AC (2015). Metabolic dependencies in ras-driven cancers., 1828-34. 10.1158/1078-0432.CCR-14-2425 PMC4400826 25878364

[157] Serchov T, Heumann R (2006). Constitutive activation of ras in neurons: implications for the regulation of the mammalian circadian clock. Chronobiol Int, 23:191-200. 10.1080/07420520500521970 16687293

[158] Giles TD (2000). Factors affecting circadian variability. Blood Press Monit, 5 Suppl 1:S3-7. 10.1097/00126097-200005001-00002 10904236

[159] Relógio A, Thomas P, Medina-Pérez P, Reischl S, Bervoets S, Gloc E, et al (2014). Ras-mediated deregulation of the circadian clock in cancer. PLoS Genet, 10:e1004338. 10.1371/journal.pgen.1004338 PMC4038477 24875049

[160] Katamune C, Koyanagi S, Shiromizu S, Matsunaga N, Shimba S, Shibata S, et al (2016). Different roles of negative and positive components of the circadian clock in oncogene-induced neoplastic transformation. The Journal of biological chemistry, 291:10541-50. 10.1074/jbc.M115.706481 PMC4865904 26961881

[161] Hashikawa K, Katamune C, Kusunose N, Matsunaga N, Koyanagi S, Ohdo S (2017). Dysfunction of the circadian transcriptional factor clock in mice resists chemical carcinogen-induced tumorigenesis. Sci Rep, 7:9995. 10.1038/s41598-017-10599-1 PMC5577256 28855649

[162] Antle MC, Tse F, Koke SJ, Sterniczuk R, Hagel K (2008). Non-photic phase shifting of the circadian clock: role of the extracellular signal-responsive kinases i/ii/mitogen-activated protein kinase pathway. The European journal of neuroscience, 28:2511-8. 10.1111/j.1460-9568.2008.06533.x 19087176

[163] Weber F, Hung H, Maurer C, Kay SA (2006). Second messenger and ras/mapk signalling pathways regulate clock/cycle-dependent transcription., 248-57. 10.1111/j.1471-4159.2006.03865.x 16805811

[164] Besing RC, Rogers CO, Paul JR, Hablitz LM, Johnson RL, McMahon LL, et al (2017). Gsk3 activity regulates rhythms in hippocampal clock gene expression and synaptic plasticity. Hippocampus, 27:890-8. 10.1002/hipo.22739 PMC5511075 28556462

[165] Besing RC, Paul JR, Hablitz LM, Rogers CO, Johnson RL, Young ME, et al (2015). Circadian rhythmicity of active gsk3 isoforms modulates molecular clock gene rhythms in the suprachiasmatic nucleus., 155-60. 10.1177/0748730415573167 PMC4586074 25724980

[166] Paul JR, Johnson RL, Jope RS, Gamble KL (2012). Disruption of circadian rhythmicity and suprachiasmatic action potential frequency in a mouse model with constitutive activation of glycogen synthase kinase 3. Neuroscience, 226:1-9. 10.1016/j.neuroscience.2012.08.047 PMC3490018 22986169

[167] Spengler ML, Kuropatwinski KK, Schumer M, Antoch MP (2009). A serine cluster mediates bmal1-dependent clock phosphorylation and degradation. Cell cycle (Georgetown, Tex.), 8:4138-46. 10.4161/cc.8.24.10273 PMC4073639 19946213

[168] Herzig S, Shaw RJ (2018). Ampk: guardian of metabolism and mitochondrial homeostasis. Nature reviews. Molecular cell biology, 19:121-35. 10.1038/nrm.2017.95 PMC5780224 28974774

[169] Sanchez-Cespedes M, Parrella P, Esteller M, Nomoto S, Trink B, Engles JM, et al (2002). Inactivation of lkb1/stk11 is a common event in adenocarcinomas of the lung. Cancer Res, 62:3659-62. 12097271

[170] Hardie DG, Alessi DR (2013). Lkb1 and ampk and the cancer-metabolism link - ten years after. BMC Biol, 11:36. 10.1186/1741-7007-11-36 PMC3626889 23587167

[171] Goodwin JM, Svensson RU, Lou HJ, Winslow MM, Turk BE, Shaw RJ (2014). An ampk-independent signaling pathway downstream of the lkb1 tumor suppressor controls snail1 and metastatic potential. Mol Cell, 55:436-50. 10.1016/j.molcel.2014.06.021 PMC4151130 25042806

[172] Xing W, Busino L, Hinds TR, Marionni ST, Saifee NH, Bush MF, et al (2013). Scf(fbxl3) ubiquitin ligase targets cryptochromes at their cofactor pocket. Nature, 496:64-8. 10.1038/nature11964 PMC3618506 23503662

[173] Lamia KA, Sachdeva UM, DiTacchio L, Williams EC, Alvarez JG, Egan DF, et al (2009). Ampk regulates the circadian clock by cryptochrome phosphorylation and degradation. Science (New York, N.Y.), 326:437-40. 10.1126/science.1172156 PMC2819106 19833968

[174] Eide EJ, Woolf MF, Kang H, Woolf P, Hurst W, Camacho F, et al (2005). Control of mammalian circadian rhythm by ckiepsilon-regulated proteasome-mediated per2 degradation. Mol Cell Biol, 25:2795-807. 10.1128/MCB.25.7.2795-2807.2005 PMC1061645 15767683

[175] Brandauer J, Vienberg SG, Andersen MA, Ringholm S, Risis S, Larsen PS, et al (2013). Amp-activated protein kinase regulates nicotinamide phosphoribosyl transferase expression in skeletal muscle. The Journal of physiology, 591:5207-20. 10.1113/jphysiol.2013.259515 PMC3810819 23918774

[176] Um JH, Yang S, Yamazaki S, Kang H, Viollet B, Foretz M, et al (2007). Activation of 5'-amp-activated kinase with diabetes drug metformin induces casein kinase iepsilon (ckiepsilon)-dependent degradation of clock protein mper2. The Journal of biological chemistry, 282:20794-8. 10.1074/jbc.C700070200 17525164

[177] Pernicova I, Korbonits M (2014). Metformin--mode of action and clinical implications for diabetes and cancer. Nature reviews. Endocrinology, 10:143-56. 10.1038/nrendo.2013.256 24393785

[178] Fabian ID, Onadim Z, Karaa E, Duncan C, Chowdhury T, Scheimberg I, et al (2018). The management of retinoblastoma. Oncogene, 37:1551-60. 10.1038/s41388-017-0050-x 29321667

[179] Kent LN, Leone G (2019). The broken cycle: e2f dysfunction in cancer. Nature reviews. Cancer, 19:326-38. 10.1038/s41568-019-0143-7 31053804

[180] Lee Y, Lahens NF, Zhang S, Bedont J, Field JM, Sehgal A (2019). G1/s cell cycle regulators mediate effects of circadian dysregulation on tumor growth and provide targets for timed anticancer treatment. PLoS Biol, 17:e3000228. 10.1371/journal.pbio.3000228 PMC6490878 31039152

[181] Chan AB, Huber A, Lamia KA (2020). Cryptochromes modulate e2f family transcription factors. Sci Rep, 10:4077. 10.1038/s41598-020-61087-y PMC7058038 32139766

[182] Chan AB, Lamia KA (2020). Cancer, hear my battle cry. J Pineal Res, 69:e12658. 10.1111/jpi.12658 PMC7572572 32291799

[183] Römer L, Klein C, Dehner A, Kessler H, Buchner J (2006). P53--a natural cancer killer: structural insights and therapeutic concepts. Angewandte Chemie (International ed. in English), 45:6440-60. 10.1002/anie.200600611 16983711

[184] Berkers CR, Maddocks ODK, Cheung EC, Mor I, Vousden KH (2013). Metabolic regulation by p53 family members. Cell Metab, 18:617-33. 10.1016/j.cmet.2013.06.019 PMC3824073 23954639

[185] Bjarnason GA, Jordan RC, Wood PA, Li Q, Lincoln DW, Sothern RB, et al (2001). Circadian expression of clock genes in human oral mucosa and skin: association with specific cell-cycle phases., 1793-801. 10.1016/S0002-9440(10)64135-1 PMC1891949 11337377

[186] Stephenson EM, Usselmann LEJ, Tergaonkar V, Virshup DM, Dallmann R (2021). Cancer clocks in tumourigenesis: the p53 pathway and beyond. Endocr Relat Cancer, 28:R95-110. 10.1530/ERC-20-0475 33638942

[187] Miki T, Matsumoto T, Zhao Z, Lee CC (2013). P53 regulates period2 expression and the circadian clock. Nat Commun, 4:2444. 10.1038/ncomms3444 PMC3798035 24051492

[188] Gotoh T, Vila-Caballer M, Liu J, Schiffhauer S, Finkielstein CV (2015). Association of the circadian factor period 2 to p53 influences p53's function in dna-damage signaling. Mol Biol Cell, 26:359-72. 10.1091/mbc.E14-05-0994 PMC4294682 25411341

[189] Gotoh T, Kim JK, Liu J, Vila-Caballer M, Stauffer PE, Tyson JJ, et al (2016). Model-driven experimental approach reveals the complex regulatory distribution of p53 by the circadian factor period 2. Proc Natl Acad Sci U S A, 113:13516-21. 10.1073/pnas.1607984113 PMC5127372 27834218

[190] Liu J, Zou X, Gotoh T, Brown AM, Jiang L, Wisdom EL, et al (2018). Distinct control of period2 degradation and circadian rhythms by the oncoprotein and ubiquitin ligase mdm2. Sci Signal, 11. 10.1126/scisignal.aau0715 30425162

[191] Gotoh T, Vila-Caballer M, Santos CS, Liu J, Yang J, Finkielstein CV (2014). The circadian factor period 2 modulates p53 stability and transcriptional activity in unstressed cells. Mol Biol Cell, 25:3081-93. 10.1091/mbc.E14-05-0993 PMC4230596 25103245

[192] Jiang W, Zhao S, Jiang X, Zhang E, Hu G, Hu B, et al (2016). The circadian clock gene bmal1 acts as a potential anti-oncogene in pancreatic cancer by activating the p53 tumor suppressor pathway. Cancer Lett, 371:314-25. 10.1016/j.canlet.2015.12.002 26683776

[193] Sun Y, Wang P, Li H, Dai J (2018). Bmal1 and clock proteins in regulating uvb-induced apoptosis and dna damage responses in human keratinocytes. J Cell Physiol, 233:9563-74. 10.1002/jcp.26859 PMC6185778 29943823

[194] Kawamura G, Hattori M, Takamatsu K, Tsukada T, Ninomiya Y, Benjamin I, et al (2018). Cooperative interaction among bmal1, hsf1, and p53 protects mammalian cells from uv stress. Commun Biol, 1:204. 10.1038/s42003-018-0209-1 PMC6250677 30480104

[195] Wang J, Morita Y, Han B, Niemann S, Löffler B, Rudolph KL (2016). Per2 induction limits lymphoid-biased haematopoietic stem cells and lymphopoiesis in the context of dna damage and ageing. Nat Cell Biol, 18:480-90. 10.1038/ncb3342 27088856

[196] Horiguchi M, Koyanagi S, Hamdan AM, Kakimoto K, Matsunaga N, Yamashita C, et al (2013). Rhythmic control of the arf-mdm2 pathway by atf4 underlies circadian accumulation of p53 in malignant cells. Cancer Res, 73:2639-49. 10.1158/0008-5472.CAN-12-2492 23580573

[197] Moser AR, Pitot HC, Dove WF (1990). A dominant mutation that predisposes to multiple intestinal neoplasia in the mouse. Science (New York, N.Y.), 247:322-4. 10.1126/science.2296722 2296722

[198] Morin PJ, Sparks AB, Korinek V, Barker N, Clevers H, Vogelstein B, et al (1997). Activation of beta-catenin-tcf signaling in colon cancer by mutations in beta-catenin or apc. Science (New York, N.Y.), 275:1787-90. 10.1126/science.275.5307.1787 9065402

[199] Wood PA, Yang X, Taber A, Oh E, Ansell C, Ayers SE, et al (2008). Period 2 mutation accelerates apcmin/+ tumorigenesis. Molecular cancer research : MCR, 6:1786-93. 10.1158/1541-7786.MCR-08-0196 PMC4136553 19010825

[200] Yang X, Wood PA, Ansell CM, Ohmori M, Oh E, Xiong Y, et al (2009). Beta-catenin induces beta-trcp-mediated per2 degradation altering circadian clock gene expression in intestinal mucosa of apcmin/+ mice. J Biochem, 145:289-97. 10.1093/jb/mvn167 19106159

[201] Zhan T, Rindtorff N, Boutros M (2017). Wnt signaling in cancer. Oncogene, 36:1461-73. 10.1038/onc.2016.304 PMC5357762 27617575

[202] Hess G, Kroegel C, Ramadori G, Rieder H, Meyer zum Büschenfelde KH (1987). Demonstration of antibodies to the surface (anti-p41) and core proteins (anti-p24) of the human immunodeficiency virus (hiv) in individuals positive for anti-hiv. Klinische Wochenschrift, 65:596-9. 10.1007/BF01726665 3498088

[203] Matsu-Ura T, Dovzhenok A, Aihara E, Rood J, Le H, Ren Y, et al (2016). Intercellular coupling of the cell cycle and circadian clock in adult stem cell culture. Mol Cell, 64:900-12. 10.1016/j.molcel.2016.10.015 PMC5423461 27867006

[204] Karpowicz P, Zhang Y, Hogenesch JB, Emery P, Perrimon N (2013). The circadian clock gates the intestinal stem cell regenerative state. Cell Rep, 3:996-1004. 10.1016/j.celrep.2013.03.016 PMC3982394 23583176

[205] Puram RV, Kowalczyk MS, de Boer CG, Schneider RK, Miller PG, McConkey M, et al (2016). Core circadian clock genes regulate leukemia stem cells in aml. Cell, 165:303-16. 10.1016/j.cell.2016.03.015 PMC4826477 27058663

[206] Janich P, Pascual G, Merlos-Suárez A, Batlle E, Ripperger J, Albrecht U, et al (2011). The circadian molecular clock creates epidermal stem cell heterogeneity. Nature, 480:209-14. 10.1038/nature10649 22080954

[207] Yang X, Downes M, Yu RT, Bookout AL, He W, Straume M, et al (2006). Nuclear receptor expression links the circadian clock to metabolism. Cell, 126:801-10. 10.1016/j.cell.2006.06.050 16923398

[208] Li S, Wang M, Ao X, Chang AK, Yang C, Zhao F, et al (2013). Clock is a substrate of sumo and sumoylation of clock upregulates the transcriptional activity of estrogen receptor-α. Oncogene, 32:4883-91. 10.1038/onc.2012.518 23160374

[209] Wang Z, Ma L, Meng Y, Fang J, Xu D, Lu Z (2023). The interplay of the circadian clock and metabolic tumorigenesis. Trends Cell Biol. 10.1016/j.tcb.2023.11.004 38061936

[210] Padilla J, Osman NM, Bissig-Choisat B, Grimm SL, Qin X, Major AM, et al (2024). Circadian dysfunction induces nafld-related human liver cancer in a mouse model. J Hepatol, 80:282-92. 10.1016/j.jhep.2023.10.018 PMC10929560 37890720

[211] Wang Y, Narasimamurthy R, Qu M, Shi N, Guo H, Xue Y, et al (2024). Circadian regulation of cancer stem cells and the tumor microenvironment during metastasis. Nat Cancer, 5:546-56. 10.1038/s43018-024-00759-4 38654103

[212] Turek FW, Joshu C, Kohsaka A, Lin E, Ivanova G, McDearmon E, et al (2005). Obesity and metabolic syndrome in circadian clock mutant mice. Science (New York, N.Y.), 308:1043-5. 10.1126/science.1108750 PMC3764501 15845877

[213] Nakahata Y, Kaluzova M, Grimaldi B, Sahar S, Hirayama J, Chen D, et al (2008). The nad+-dependent deacetylase sirt1 modulates clock-mediated chromatin remodeling and circadian control. Cell, 134:329-40. 10.1016/j.cell.2008.07.002 PMC3526943 18662547

[214] Alenghat T, Meyers K, Mullican SE, Leitner K, Adeniji-Adele A, Avila J, et al (2008). Nuclear receptor corepressor and histone deacetylase 3 govern circadian metabolic physiology. Nature, 456:997-1000. 10.1038/nature07541 PMC2742159 19037247

[215] Wu J, Jing X, Du Q, Sun X, Holgersson K, Gao J, et al (2023). Disruption of the clock component bmal1 in mice promotes cancer metastasis through the pai-1-tgf-β-myocaf-dependent mechanism. Advanced science (Weinheim, Baden-Wurttemberg, Germany), 10:e2301505. 10.1002/advs.202301505 PMC10460897 37330661

[216] Finley LWS (2023). What is cancer metabolism? Cell, 186:1670-88. 10.1016/j.cell.2023.01.038 PMC10106389 36858045

[217] Peek CB, Affinati AH, Ramsey KM, Kuo H, Yu W, Sena LA, et al (2013). Circadian clock nad+ cycle drives mitochondrial oxidative metabolism in mice. Science (New York, N.Y.), 342:1243417. 10.1126/science.1243417 PMC3963134 24051248

[218] Scheer FAJL, Hilton MF, Mantzoros CS, Shea SA (2009). Adverse metabolic and cardiovascular consequences of circadian misalignment. Proc Natl Acad Sci U S A, 106:4453-8. 10.1073/pnas.0808180106 PMC2657421 19255424

[219] He L, Fan Y, Zhang Y, Tu T, Zhang Q, Yuan F, et al (2022). Single-cell transcriptomic analysis reveals circadian rhythm disruption associated with poor prognosis and drug-resistance in lung adenocarcinoma. J Pineal Res, 73:e12803. 10.1111/jpi.12803 35436363

[220] Cheng Y, Yao J, Fang Q, Chen B, Zang G (2022). A circadian rhythm-related biomarker for predicting prognosis and immunotherapy efficacy in lung adenocarcinoma. Aging, 14:9617-31. 10.18632/aging.204411 PMC9792196 36455876

[221] Zhu Y, Li X, Wang L, Hong X, Yang J (2022). Metabolic reprogramming and crosstalk of cancer-related fibroblasts and immune cells in the tumor microenvironment. Front Endocrinol (Lausanne), 13:988295. 10.3389/fendo.2022.988295 PMC9421293 36046791

[222] Chun SK, Fortin BM, Fellows RC, Habowski AN, Verlande A, Song WA, et al (2022). Disruption of the circadian clock drives apc loss of heterozygosity to accelerate colorectal cancer. Sci Adv, 8:eabo2389. 10.1126/sciadv.abo2389 PMC9365282 35947664

[223] Muscogiuri G, Barrea L, Aprano S, Framondi L, Di Matteo R, Altieri B, et al (2021). Chronotype and cardio metabolic health in obesity: does nutrition matter? Int J Food Sci Nutr, 72:892-900. 10.1080/09637486.2021.1885017 33759693

[224] Ekiz Erim S, Sert H (2023). The relationship between chronotype and obesity: a systematic review. Chronobiol Int, 40:529-41. 10.1080/07420528.2023.2180385 36803075

[225] Trevellin E, Bettini S, Pilatone A, Vettor R, Milan G (2023). Obesity, the adipose organ and cancer in humans: association or causation? Biomedicines, 11. 10.3390/biomedicines11051319 PMC10215824 37238992

[226] Kelleher FC, Rao A, Maguire A (2014). Circadian molecular clocks and cancer. Cancer Lett, 342:9-18. 10.1016/j.canlet.2013.09.040 24099911

[227] Miro C, Docimo A, Barrea L, Verde L, Cernea S, Sojat AS, et al (2023). "Time" for obesity-related cancer: the role of the circadian rhythm in cancer pathogenesis and treatment. Semin Cancer Biol, 91:99-109. 10.1016/j.semcancer.2023.03.003 36893964

[228] Montaigne D, Butruille L, Staels B (2021). Ppar control of metabolism and cardiovascular functions. Nature reviews. Cardiology, 18:809-23. 10.1038/s41569-021-00569-6 34127848

[229] Wang L, Waltenberger B, Pferschy-Wenzig E, Blunder M, Liu X, Malainer C, et al (2014). Natural product agonists of peroxisome proliferator-activated receptor gamma (pparγ): a review. Biochem Pharmacol, 92:73-89. 10.1016/j.bcp.2014.07.018 PMC4212005 25083916

[230] Chang H, Guarente L (2014). Sirt1 and other sirtuins in metabolism. Trends in endocrinology and metabolism: TEM, 25:138-45. 10.1016/j.tem.2013.12.001 PMC3943707 24388149

[231] Zhu M, Wei C, Wang H, Han S, Cai L, Li X, et al (2023). Sirt1 mediated gastric cancer progression under glucose deprivation through the foxo1-rab7-autophagy axis. Front Oncol, 13:1175151. 10.3389/fonc.2023.1175151 PMC10244632 37293593

[232] Lo M, Chen J, Kuo Y, Chen W, Lee H, Wang S (2019). Camptothecin activates sirt1 to promote lipid catabolism through ampk/foxo1/atgl pathway in c(2)c(12) myogenic cells. Arch Pharm Res, 42:672-83. 10.1007/s12272-019-01155-8 31020545

[233] Li M, Cai Y, Chen X, Zhang L, Jiang Z, Yu Q (2022). Tamoxifen induced hepatic steatosis in high-fat feeding rats through sirt1-foxo1 suppression and lxr-srebp1c activation. Toxicol Res (Camb), 11:673-82. 10.1093/toxres/tfac043 PMC9424708 36051666

[234] Imai S, Armstrong CM, Kaeberlein M, Guarente L (2000). Transcriptional silencing and longevity protein sir2 is an nad-dependent histone deacetylase. Nature, 403:795-800. 10.1038/35001622 10693811

[235] Ramsey KM, Yoshino J, Brace CS, Abrassart D, Kobayashi Y, Marcheva B, et al (2009). Circadian clock feedback cycle through nampt-mediated nad+ biosynthesis. Science (New York, N.Y.), 324:651-4. 10.1126/science.1171641 PMC2738420 19299583

[236] Nakahata Y, Sahar S, Astarita G, Kaluzova M, Sassone-Corsi P (2009). Circadian control of the nad+ salvage pathway by clock-sirt1. Science (New York, N.Y.), 324:654-7. 10.1126/science.1170803 PMC6501775 19286518

[237] Kolthur-Seetharam U, Dantzer F, McBurney MW, de Murcia G, Sassone-Corsi P (2006). Control of aif-mediated cell death by the functional interplay of sirt1 and parp-1 in response to dna damage. Cell cycle (Georgetown, Tex.), 5:873-7. 10.4161/cc.5.8.2690 16628003

[238] Garten A, Petzold S, Körner A, Imai S, Kiess W (2009). Nampt: linking nad biology, metabolism and cancer. Trends in endocrinology and metabolism: TEM, 20:130-8. 10.1016/j.tem.2008.10.004 PMC2738422 19109034

[239] Hasmann M, Schemainda I (2003). Fk866, a highly specific noncompetitive inhibitor of nicotinamide phosphoribosyltransferase, represents a novel mechanism for induction of tumor cell apoptosis. Cancer Res, 63:7436-42. 14612543

[240] Gorman MR (2020). Temporal organization of pineal melatonin signaling in mammals. Mol Cell Endocrinol, 503:110687. 10.1016/j.mce.2019.110687 31866317

[241] Pevet P, Challet E (2011). Melatonin: both master clock output and internal time-giver in the circadian clocks network. Journal of physiology, Paris, 105:170-82. 10.1016/j.jphysparis.2011.07.001 21914478

[242] Owino S, Buonfiglio DDC, Tchio C, Tosini G (2019). Melatonin signaling a key regulator of glucose homeostasis and energy metabolism. Front Endocrinol (Lausanne), 10:488. 10.3389/fendo.2019.00488 PMC6651071 31379753

[243] Owino S, Contreras-Alcantara S, Baba K, Tosini G (2016). Melatonin signaling controls the daily rhythm in blood glucose levels independent of peripheral clocks. PLoS One, 11:e0148214. 10.1371/journal.pone.0148214 PMC4732609 26824606

[244] Contreras-Alcantara S, Baba K, Tosini G (2010). Removal of melatonin receptor type 1 induces insulin resistance in the mouse. Obesity (Silver Spring, Md.), 18:1861-3. 10.1038/oby.2010.24 PMC2929321 20168308

[245] Dauchy RT, Blask DE, Sauer LA, Brainard GC, Krause JA (1999). Dim light during darkness stimulates tumor progression by enhancing tumor fatty acid uptake and metabolism. Cancer Lett, 144:131-6. 10.1016/s0304-3835(99)00207-4 10529012

[246] Mhatre MC, Shah PN, Juneja HS (1984). Effect of varying photoperiods on mammary morphology, dna synthesis, and hormone profile in female rats., 1411-6. 6427503

[247] Talib WH (2018). Melatonin and cancer hallmarks. Molecules (Basel, Switzerland), 23. 10.3390/molecules23030518 PMC6017729 29495398

[248] Li Y, Li S, Zhou Y, Meng X, Zhang J, Xu D, et al (2017). Melatonin for the prevention and treatment of cancer. Oncotarget, 8:39896-921. 10.18632/oncotarget.16379 PMC5503661 28415828

[249] Shah PN, Mhatre MC, Kothari LS (1984). Effect of melatonin on mammary carcinogenesis in intact and pinealectomized rats in varying photoperiods. Cancer Res, 44:3403-7. 6430548

[250] Ishida A, Mutoh T, Ueyama T, Bando H, Masubuchi S, Nakahara D, et al (2005). Light activates the adrenal gland: timing of gene expression and glucocorticoid release. Cell Metab, 2:297-307. 10.1016/j.cmet.2005.09.009 16271530

[251] Kiessling S, Sollars PJ, Pickard GE (2014). Light stimulates the mouse adrenal through a retinohypothalamic pathway independent of an effect on the clock in the suprachiasmatic nucleus. PLoS One, 9:e92959. 10.1371/journal.pone.0092959 PMC3962469 24658072

[252] Oster H, Damerow S, Kiessling S, Jakubcakova V, Abraham D, Tian J, et al (2006). The circadian rhythm of glucocorticoids is regulated by a gating mechanism residing in the adrenal cortical clock. Cell Metab, 4:163-73. 10.1016/j.cmet.2006.07.002 16890544

[253] Son GH, Chung S, Choe HK, Kim H, Baik S, Lee H, et al (2008). Adrenal peripheral clock controls the autonomous circadian rhythm of glucocorticoid by causing rhythmic steroid production. Proc Natl Acad Sci U S A, 105:20970-5. 10.1073/pnas.0806962106 PMC2634940 19091946

[254] Filipski E, King VM, Li X, Granda TG, Mormont M, Claustrat B, et al (2003). Disruption of circadian coordination accelerates malignant growth in mice. Pathologie-biologie, 51:216-9. 10.1016/s0369-8114(03)00034-8 12852994

[255] Moreno-Smith M, Lutgendorf SK, Sood AK (2010). Impact of stress on cancer metastasis. Future oncology (London, England), 6:1863-81. 10.2217/fon.10.142 PMC3037818 21142861

[256] Yang H, Xia L, Chen J, Zhang S, Martin V, Li Q, et al (2019). Stress-glucocorticoid-tsc22d3 axis compromises therapy-induced antitumor immunity. Nat Med, 25:1428-41. 10.1038/s41591-019-0566-4 31501614

[257] Shimba A, Cui G, Tani-Ichi S, Ogawa M, Abe S, Okazaki F, et al (2018). Glucocorticoids drive diurnal oscillations in t cell distribution and responses by inducing interleukin-7 receptor and cxcr4. Immunity, 48:286-98. 10.1016/j.immuni.2018.01.004 29396162

[258] Partch CL, Sancar A (2005). Photochemistry and photobiology of cryptochrome blue-light photopigments: the search for a photocycle. Photochem Photobiol, 81:1291-304. 10.1562/2005-07-08-IR-607 16164372

[259] Parkin J, Cohen B (2001). An overview of the immune system. Lancet (London, England), 357:1777-89. 10.1016/S0140-6736(00)04904-7 11403834

[260] Carroll RG, Timmons GA, Cervantes-Silva MP, Kennedy OD, Curtis AM (2019). Immunometabolism around the clock. Trends Mol Med, 25:612-25. 10.1016/j.molmed.2019.04.013 31153819

[261] Scheiermann C, Kunisaki Y, Frenette PS (2013). Circadian control of the immune system. Nature reviews. Immunology, 13:190-8. 10.1038/nri3386 PMC4090048 23391992

[262] Early JO, Curtis AM (2016). Immunometabolism: is it under the eye of the clock? Semin Immunol, 28:478-90. 10.1016/j.smim.2016.10.006 27884543

[263] Kovac J, Husse J, Oster H (2009). A time to fast, a time to feast: the crosstalk between metabolism and the circadian clock. Mol Cells, 28:75-80. 10.1007/s10059-009-0113-0 19714310

[264] Wang C, Zeng Q, Gül ZM, Wang S, Pick R, Cheng P, et al (2024). Circadian tumor infiltration and function of cd8(+) t cells dictate immunotherapy efficacy. Cell, 187:2690-702. 10.1016/j.cell.2024.04.015 38723627

[265] Zeng Y, Guo Z, Wu M, Chen F, Chen L (2024). Circadian rhythm regulates the function of immune cells and participates in the development of tumors. Cell death discovery, 10:199. 10.1038/s41420-024-01960-1 PMC11055927 38678017

[266] Zhang X, Pant SM, Ritch CC, Tang H, Shao H, Dweep H, et al (2024). Cell state dependent effects of bmal1 on melanoma immunity and tumorigenicity. Nat Commun, 15:633. 10.1038/s41467-024-44778-2 PMC10799901 38245503

[267] Nilsonne G, Lekander M, Åkerstedt T, Axelsson J, Ingre M (2016). Diurnal variation of circulating interleukin-6 in humans: a meta-analysis. PLoS One, 11:e0165799. 10.1371/journal.pone.0165799 PMC5104468 27832117

[268] Liu J, Wang C, Cheng T, Rixiati Y, Ji C, Deng M, et al (2021). Circadian clock disruption suppresses pdl1(+) intraepithelial b cells in experimental colitis and colitis-associated colorectal cancer. Cell Mol Gastroenterol Hepatol, 12:251-76. 10.1016/j.jcmgh.2021.02.008 PMC8141473 33652118

[269] Logan RW, Zhang C, Murugan S, O'Connell S, Levitt D, Rosenwasser AM, et al (2012). Chronic shift-lag alters the circadian clock of nk cells and promotes lung cancer growth in rats. Journal of immunology (Baltimore, Md. : 1950), 188:2583-91. 10.4049/jimmunol.1102715 PMC3294088 22308312

[270] Cheng W, Lam K, Li X, Kong AP, Cheung PC (2021). Circadian disruption-induced metabolic syndrome in mice is ameliorated by oat β-glucan mediated by gut microbiota. Carbohydr Polym, 267:118216. 10.1016/j.carbpol.2021.118216 34119170

[271] Wu Y, Tao B, Zhang T, Fan Y, Mao R (2019). Pan-cancer analysis reveals disrupted circadian clock associates with t cell exhaustion. Front Immunol, 10:2451. 10.3389/fimmu.2019.02451 PMC6821711 31708917

[272] Fonken LK, Weil ZM, Nelson RJ (2013). Mice exposed to dim light at night exaggerate inflammatory responses to lipopolysaccharide. Brain, behavior, and immunity, 34:159-63. 10.1016/j.bbi.2013.08.011 24012645

[273] Oakley RH, Ramamoorthy S, Foley JF, Busada JT, Lu NZ, Cidlowski JA (2018). Glucocorticoid receptor isoform-specific regulation of development, circadian rhythm, and inflammation in mice. FASEB journal : official publication of the Federation of American Societies for Experimental Biology, 32:5258-71. 10.1096/fj.201701153R PMC6133704 29672221

[274] Ince LM, Zhang Z, Beesley S, Vonslow RM, Saer BR, Matthews LC, et al (2019). Circadian variation in pulmonary inflammatory responses is independent of rhythmic glucocorticoid signaling in airway epithelial cells. FASEB journal : official publication of the Federation of American Societies for Experimental Biology, 33:126-39. 10.1096/fj.201800026RR PMC6355062 29965797

[275] Gibbs JE, Blaikley J, Beesley S, Matthews L, Simpson KD, Boyce SH, et al (2012). The nuclear receptor rev-erbα mediates circadian regulation of innate immunity through selective regulation of inflammatory cytokines. Proc Natl Acad Sci U S A, 109:582-7. 10.1073/pnas.1106750109 PMC3258648 22184247

[276] Yamamura Y, Yano I, Kudo T, Shibata S (2010). Time-dependent inhibitory effect of lipopolysaccharide injection on per1 and per2 gene expression in the mouse heart and liver. Chronobiol Int, 27:213-32. 10.3109/07420521003769111 20370466

[277] Hashiramoto A, Yamane T, Tsumiyama K, Yoshida K, Komai K, Yamada H, et al (2010). Mammalian clock gene cryptochrome regulates arthritis via proinflammatory cytokine tnf-alpha. Journal of immunology (Baltimore, Md. : 1950), 184:1560-5. 10.4049/jimmunol.0903284 20042581

[278] Sancar A, Van Gelder RN (2021). Clocks, cancer, and chronochemotherapy. Science (New York, N.Y.), 371. 10.1126/science.abb0738 33384351

[279] Yang Y, Lindsey-Boltz LA, Vaughn CM, Selby CP, Cao X, Liu Z, et al (2021). Circadian clock, carcinogenesis, chronochemotherapy connections. The Journal of biological chemistry, 297:101068. 10.1016/j.jbc.2021.101068 PMC8403766 34375638

[280] Ballesta A, Innominato PF, Dallmann R, Rand DA, Lévi FA (2017). Systems chronotherapeutics. Pharmacol Rev, 69:161-99. 10.1124/pr.116.013441 PMC5394920 28351863

[281] Lévi F, Altinok A, Clairambault J, Goldbeter A (2008). Implications of circadian clocks for the rhythmic delivery of cancer therapeutics. Philosophical transactions. Series A, Mathematical, physical, and engineering sciences, 366:3575-98. 10.1098/rsta.2008.0114 18644767

[282] Ozturk N, Ozturk D, Kavakli IH, Okyar A (2017). Molecular aspects of circadian pharmacology and relevance for cancer chronotherapy. International journal of molecular sciences, 18. 10.3390/ijms18102168 PMC5666849 29039812

[283] Bernard S, Cajavec Bernard B, Lévi F, Herzel H (2010). Tumor growth rate determines the timing of optimal chronomodulated treatment schedules. PLoS Comput Biol, 6:e1000712. 10.1371/journal.pcbi.1000712 PMC2841621 20333244

[284] Hesse J, Martinelli J, Aboumanify O, Ballesta A, Relógio A (2021). A mathematical model of the circadian clock and drug pharmacology to optimize irinotecan administration timing in colorectal cancer. Comput Struct Biotechnol J, 19:5170-83. 10.1016/j.csbj.2021.08.051 PMC8477139 34630937

[285] Dulong S, Ballesta A, Okyar A, Lévi F (2015). Identification of circadian determinants of cancer chronotherapy through in vitro chronopharmacology and mathematical modeling. Mol Cancer Ther, 14:2154-64. 10.1158/1535-7163.MCT-15-0129 26141947

[286] Bajetta E, Pietrantonio F, Buzzoni R, Ferrario E, Valvo F, Mariani L, et al (2014). Chronomodulated capecitabine and adjuvant radiation in intermediate-risk to high-risk rectal cancer: a phase ii study., 545-9. 10.1097/COC.0b013e31827ecd1d 23428953

[287] Akgun Z, Saglam S, Yucel S, Gural Z, Balik E, Cipe G, et al (2014). Neoadjuvant chronomodulated capecitabine with radiotherapy in rectal cancer: a phase ii brunch regimen study., 751-6. 10.1007/s00280-014-2558-x 25102935

[288] Dallmann R, Okyar A, Lévi F (2016). Dosing-time makes the poison: circadian regulation and pharmacotherapy. Trends Mol Med, 22:430-45. 10.1016/j.molmed.2016.03.004 27066876

[289] Dong D, Yang D, Lin L, Wang S, Wu B (2020). Circadian rhythm in pharmacokinetics and its relevance to chronotherapy. Biochem Pharmacol, 178:114045. 10.1016/j.bcp.2020.114045 32446886

[290] Lévi FA, Boige V, Hebbar M, Smith D, Lepère C, Focan C, et al (2016). Conversion to resection of liver metastases from colorectal cancer with hepatic artery infusion of combined chemotherapy and systemic cetuximab in multicenter trial optiliv., 267-74. 10.1093/annonc/mdv548 26578731

[291] Bouchahda M, Adam R, Giacchetti S, Castaing D, Brezault-Bonnet C, Hauteville D, et al (2009). Rescue chemotherapy using multidrug chronomodulated hepatic arterial infusion for patients with heavily pretreated metastatic colorectal cancer., 4990-9. 10.1002/cncr.24549 19637365

[292] Lévi F, Karaboué A, Etienne-Grimaldi M, Paintaud G, Focan C, Innominato P, et al (2017). Pharmacokinetics of irinotecan, oxaliplatin and 5-fluorouracil during hepatic artery chronomodulated infusion: a translational european optiliv study., 165-77. 10.1007/s40262-016-0431-2 27393140

[293] Hrushesky WJ (1985). Circadian timing of cancer chemotherapy., 73-5. 10.1126/science.3883493 3883493

[294] Hrushesky WJ, Bjarnason GA (1993). Circadian cancer therapy. Journal of clinical oncology : official journal of the American Society of Clinical Oncology, 11:1403-17. 10.1200/JCO.1993.11.7.1403 8315438

[295] Lévi F, Benavides M, Chevelle C, Le Saunier F, Bailleul F, Misset JL, et al (1990). Chemotherapy of advanced ovarian cancer with 4'-o-tetrahydropyranyl doxorubicin and cisplatin: a randomized phase ii trial with an evaluation of circadian timing and dose-intensity., 705-14. 10.1200/JCO.1990.8.4.705 2179481

[296] Giacchetti S, Bjarnason G, Garufi C, Genet D, Iacobelli S, Tampellini M, et al (2006). Phase iii trial comparing 4-day chronomodulated therapy versus 2-day conventional delivery of fluorouracil, leucovorin, and oxaliplatin as first-line chemotherapy of metastatic colorectal cancer: the european organisation for research and treatment of cancer chronotherapy group., 3562-9. 10.1200/JCO.2006.06.1440 16877722

[297] Giacchetti S, Dugué PA, Innominato PF, Bjarnason GA, Focan C, Garufi C, et al (2012). Sex moderates circadian chemotherapy effects on survival of patients with metastatic colorectal cancer: a meta-analysis., 3110-6. 10.1093/annonc/mds148 22745214

[298] Hirota T, Lee JW, St John PC, Sawa M, Iwaisako K, Noguchi T, et al (2012). Identification of small molecule activators of cryptochrome. Science (New York, N.Y.), 337:1094-7. 10.1126/science.1223710 PMC3589997 22798407

[299] Solt LA, Wang Y, Banerjee S, Hughes T, Kojetin DJ, Lundasen T, et al (2012). Regulation of circadian behaviour and metabolism by synthetic rev-erb agonists. Nature, 485:62-8. 10.1038/nature11030 PMC3343186 22460951

[300] Ribeiro RFN, Cavadas C, Silva MMC (2021). Small-molecule modulators of the circadian clock: pharmacological potentials in circadian-related diseases. Drug Discov Today, 26:1620-41. 10.1016/j.drudis.2021.03.015 33781946

[301] Hirota T, Lee JW, St John PC, Sawa M, Iwaisako K, Noguchi T, et al (2012). Identification of small molecule activators of cryptochrome. Science (New York, N.Y.), 337:1094-7. 10.1126/science.1223710 PMC3589997 22798407

[302] Humphries PS, Bersot R, Kincaid J, Mabery E, McCluskie K, Park T, et al (2016). Carbazole-containing sulfonamides and sulfamides: discovery of cryptochrome modulators as antidiabetic agents. Bioorg Med Chem Lett, 26:757-60. 10.1016/j.bmcl.2015.12.102 26778255

[303] Chun SK, Chung S, Kim H, Lee JH, Jang J, Kim J, et al (2015). A synthetic cryptochrome inhibitor induces anti-proliferative effects and increases chemosensitivity in human breast cancer cells. Biochem Biophys Res Commun, 467:441-6. 10.1016/j.bbrc.2015.09.103 26407844

[304] Wang J, Zou JX, Xue X, Cai D, Zhang Y, Duan Z, et al (2016). Ror-γ drives androgen receptor expression and represents a therapeutic target in castration-resistant prostate cancer. Nat Med, 22:488-96. 10.1038/nm.4070 PMC5030109 27019329

[305] Lytle NK, Ferguson LP, Rajbhandari N, Gilroy K, Fox RG, Deshpande A, et al (2019). A multiscale map of the stem cell state in pancreatic adenocarcinoma. Cell, 177:572-86. 10.1016/j.cell.2019.03.010 PMC6711371 30955884

[306] Hu X, Liu X, Moisan J, Wang Y, Lesch CA, Spooner C, et al (2016). Synthetic rorγ agonists regulate multiple pathways to enhance antitumor immunity. Oncoimmunology, 5:e1254854. 10.1080/2162402X.2016.1254854 PMC5215247 28123897

[307] Jetten AM, Cook DN (2020). (Inverse) agonists of retinoic acid-related orphan receptor γ: regulation of immune responses, inflammation, and autoimmune disease. Annu Rev Pharmacol Toxicol, 60:371-90. 10.1146/annurev-pharmtox-010919-023711 PMC6952538 31386594

[308] Liu X, Zawidzka EM, Li H, Lesch CA, Dunbar J, Bousley D, et al (2019). Rorγ agonists enhance the sustained antitumor activity through intrinsic tc17 cytotoxicity and tc1 recruitment. Cancer Immunol Res, 7:1054-63. 10.1158/2326-6066.CIR-18-0714 31064778

[309] Chang MR, Dharmarajan V, Doebelin C, Garcia-Ordonez RD, Novick SJ, Kuruvilla DS, et al (2016). Synthetic rorγt agonists enhance protective immunity. ACS Chem Biol, 11:1012-8. 10.1021/acschembio.5b00899 PMC5178133 26785144

[310] Cash E, Sephton S, Woolley C, Elbehi AM, R I A, Ekine-Afolabi B, et al (2021). The role of the circadian clock in cancer hallmark acquisition and immune-based cancer therapeutics. Journal of experimental & clinical cancer research : CR, 40:119. 10.1186/s13046-021-01919-5 PMC8017624 33794967

[311] Ashrafizadeh M, Zarrabi A, Saberifar S, Hashemi F, Hushmandi K, Hashemi F, et al (2020). Nobiletin in cancer therapy: how this plant derived-natural compound targets various oncogene and onco-suppressor pathways. Biomedicines, 8. 10.3390/biomedicines8050110 PMC7277899 32380783

[312] Cao X, Yang Y, Selby CP, Liu Z, Sancar A (2021). Molecular mechanism of the repressive phase of the mammalian circadian clock. Proc Natl Acad Sci U S A, 118. 10.1073/pnas.2021174118 PMC7812753 33443219

[313] Ye R, Selby CP, Chiou Y, Ozkan-Dagliyan I, Gaddameedhi S, Sancar A (2014). Dual modes of clock:bmal1 inhibition mediated by cryptochrome and period proteins in the mammalian circadian clock. Genes Dev, 28:1989-98. 10.1101/gad.249417.114 PMC4173159 25228643

[314] Chiou Y, Yang Y, Rashid N, Ye R, Selby CP, Sancar A (2016). Mammalian period represses and de-represses transcription by displacing clock-bmal1 from promoters in a cryptochrome-dependent manner. Proc Natl Acad Sci U S A, 113:E6072-9. 10.1073/pnas.1612917113 PMC5068302 27688755

[315] Parico GCG, Perez I, Fribourgh JL, Hernandez BN, Lee H, Partch CL (2020). The human cry1 tail controls circadian timing by regulating its association with clock:bmal1. Proc Natl Acad Sci U S A, 117:27971-9. 10.1073/pnas.1920653117 PMC7668087 33106415

[316] Dong Z, Zhang G, Qu M, Gimple RC, Wu Q, Qiu Z, et al (2019). Targeting glioblastoma stem cells through disruption of the circadian clock. Cancer Discov, 9:1556-73. 10.1158/2159-8290.CD-19-0215 PMC6983300 31455674

[317] Zhang Y, Fang B, Emmett MJ, Damle M, Sun Z, Feng D, et al (2015). Gene regulation. Discrete functions of nuclear receptor rev-erbα couple metabolism to the clock. Science (New York, N.Y.), 348:1488-92. 10.1126/science.aab3021 PMC4613749 26044300

[318] Kojetin DJ, Burris TP (2014). Rev-erb and ror nuclear receptors as drug targets. Nature reviews. Drug discovery, 13:197-216. 10.1038/nrd4100 PMC4865262 24577401

[319] Sulli G, Rommel A, Wang X, Kolar MJ, Puca F, Saghatelian A, et al (2018). Pharmacological activation of rev-erbs is lethal in cancer and oncogene-induced senescence. Nature, 553:351-5. 10.1038/nature25170 PMC5924733 29320480

[320] Wagner PM, Monjes NM, Guido ME (2019). Chemotherapeutic effect of sr9009, a rev-erb agonist, on the human glioblastoma t98g cells. ASN Neuro, 11:1664469993. 10.1177/1759091419892713 PMC6909277 31825658

[321] Shen W, Zhang W, Ye W, Wang H, Zhang Q, Shen J, et al (2020). Sr9009 induces a rev-erb dependent anti-small-cell lung cancer effect through inhibition of autophagy. Theranostics, 10:4466-80. 10.7150/thno.42478 PMC7150483 32292508

[322] Trump RP, Bresciani S, Cooper AWJ, Tellam JP, Wojno J, Blaikley J, et al (2013). Optimized chemical probes for rev-erbα. J Med Chem, 56:4729-37. 10.1021/jm400458q PMC4347663 23656296

[323] Narasimamurthy R, Virshup DM (2021). The phosphorylation switch that regulates ticking of the circadian clock. Mol Cell, 81:1133-46. 10.1016/j.molcel.2021.01.006 33545069

[324] Di Maira G, Gentilini A, Pastore M, Caligiuri A, Piombanti B, Raggi C, et al (2019). The protein kinase ck2 contributes to the malignant phenotype of cholangiocarcinoma cells. Oncogenesis, 8:61. 10.1038/s41389-019-0171-x PMC6805921 31641101

[325] Monastyrskyi A, Nilchan N, Quereda V, Noguchi Y, Ruiz C, Grant W, et al (2018). Development of dual casein kinase 1δ/1ε (ck1δ/ε) inhibitors for treatment of breast cancer. Bioorg Med Chem, 26:590-602. 10.1016/j.bmc.2017.12.020 PMC5803353 29289448

[326] Liu M, Hu Y, Lu S, Lu M, Li J, Chang H, et al (2020). Ic261, a specific inhibitor of ck1δ/ε, promotes aerobic glycolysis through p53-dependent mechanisms in colon cancer. Int J Biol Sci, 16:882-92. 10.7150/ijbs.40960 PMC7019134 32071557

[327] Oshima T, Niwa Y, Kuwata K, Srivastava A, Hyoda T, Tsuchiya Y, et al (2019). Cell-based screen identifies a new potent and highly selective ck2 inhibitor for modulation of circadian rhythms and cancer cell growth. Sci Adv, 5:eaau9060. 10.1126/sciadv.aau9060 PMC6357737 30746467

[328] Gowda C, Sachdev M, Muthusami S, Kapadia M, Petrovic-Dovat L, Hartman M, et al (2017). Casein kinase ii (ck2) as a therapeutic target for hematological malignancies. Curr Pharm Des, 23:95-107. 10.2174/1381612822666161006154311 27719640

[329] Janovská P, Normant E, Miskin H, Bryja V (2020). Targeting casein kinase 1 (ck1) in hematological cancers. International journal of molecular sciences, 21. 10.3390/ijms21239026 PMC7730698 33261128

[330] Najafi M, Salehi E, Farhood B, Nashtaei MS, Hashemi Goradel N, Khanlarkhani N, et al (2019). Adjuvant chemotherapy with melatonin for targeting human cancers: a review. J Cell Physiol, 234:2356-72. 10.1002/jcp.27259 30192001

[331] Wang Y, Jin B, Ai F, Duan C, Lu Y, Dong T, et al (2012). The efficacy and safety of melatonin in concurrent chemotherapy or radiotherapy for solid tumors: a meta-analysis of randomized controlled trials. Cancer Chemother Pharmacol, 69:1213-20. 10.1007/s00280-012-1828-8 22271210

[332] Rodriguez-Garcia A, Mayo JC, Hevia D, Quiros-Gonzalez I, Navarro M, Sainz RM (2013). Phenotypic changes caused by melatonin increased sensitivity of prostate cancer cells to cytokine-induced apoptosis. J Pineal Res, 54:33-45. 10.1111/j.1600-079X.2012.01017.x 22738066

[333] Lissoni P (2007). Biochemotherapy with standard chemotherapies plus the pineal hormone melatonin in the treatment of advanced solid neoplasms. Pathologie-biologie, 55:201-4. 10.1016/j.patbio.2006.12.025 17446010

[334] Shimba A, Ikuta K (2020). Glucocorticoids regulate circadian rhythm of innate and adaptive immunity. Front Immunol, 11:2143. 10.3389/fimmu.2020.02143 PMC7533542 33072078

[335] Pufall MA (2015). Glucocorticoids and cancer. Adv Exp Med Biol, 872:315-33. 10.1007/978-1-4939-2895-8_14 PMC5546099 26216001

[336] Kiessling S, Beaulieu-Laroche L, Blum ID, Landgraf D, Welsh DK, Storch K, et al (2017). Enhancing circadian clock function in cancer cells inhibits tumor growth. BMC Biol, 15:13. 10.1186/s12915-017-0349-7 PMC5310078 28196531

